# Multilevel Growth Curve Analyses of Treatment Effects of a Web-Based Intervention for Stress Reduction: Randomized Controlled Trial

**DOI:** 10.2196/jmir.2570

**Published:** 2013-04-22

**Authors:** Filip Drozd, Sabine Raeder, Pål Kraft, Cato Alexander Bjørkli

**Affiliations:** ^1^Research and DevelopmentChangetech ASOsloNorway; ^2^Department of PsychologyUniversity of OsloOsloNorway

**Keywords:** stress, multilevel modeling, randomized controlled trial, mindfulness, procrastination, multiple mediation, multiple moderation, Web

## Abstract

**Background:**

Stress is commonly experienced by many people and it is a contributing factor to many mental and physical health conditions, However, few efforts have been made to develop and test the effects of interventions for stress.

**Objective:**

The aim of this study was to examine the effects of a Web-based stress-reduction intervention on stress, investigate mindfulness and procrastination as potential mediators of any treatment effects, and test whether the intervention is equally effective for females as males, all ages, and all levels of education.

**Methods:**

We employed a randomized controlled trial in this study. Participants were recruited online via Facebook and randomly assigned to either the stress intervention or a control condition. The Web-based stress intervention was fully automated and consisted of 13 sessions over 1 month. The controls were informed that they would get access to the intervention after the final data collection. Data were collected at baseline and at 1, 2, and 6 months after intervention onset by means of online questionnaires. Outcomes were stress, mindfulness, and procrastination, which were all measured at every measurement occasion.

**Results:**

A total of 259 participants were included and were allocated to either the stress intervention (n=126) or the control condition (n=133). Participants in the intervention and control group were comparable at baseline; however, results revealed that participants in the stress intervention followed a statistically different (ie, cubic) developmental trajectory in stress levels over time compared to the controls. A growth curve analysis showed that participants in the stress intervention (unstandardized beta coefficient [B]=–3.45, *P*=.008) recovered more quickly compared to the control group (B=–0.81, *P*=.34) from baseline to 1 month. Although participants in the stress intervention did show increases in stress levels during the study period (B=2.23, *P*=.008), long-term stress levels did decrease again toward study end at 6 months (B=–0.28, *P*=.009). Stress levels in the control group, however, remained largely unchanged after 1 month (B=0.29, *P*=.61) and toward 6 months (B=–0.03, *P*=.67). Mediation analyses showed nonlinear (ie, cubic) specific indirect effects of mindfulness and a linear specific indirect effect of procrastination on stress. In simple terms, the intervention increased mindfulness and decreased procrastination, which was related to lower stress levels. Finally, the effect of the stress intervention was independent of participants’ gender, age, or education.

**Conclusions:**

The results from this randomized controlled trial suggest that a Web-based intervention can reduce levels of stress in a normal population and that both mindfulness and procrastination may be important components included in future eHealth interventions for stress.

**Trial Registration:**

International Standard Randomized Controlled Trial Number (ISRCTN): 25619675; http://controlled-trials.com/ISRCTN25619675 (Archived by Webcite at http://www.webcitation.org/6FxB1gOKY)

## Introduction

Symptoms of stress, such as fatigue, mood changes, and muscle pain, are very common in the general population. More than 90% of the Norwegian population reported having several such symptoms during the past 30 days [1]. According to an international survey, approximately 75% of the general population in developed countries report feeling stressed on a daily basis [2]. Furthermore, the most recent Stress in America survey conducted by the American Psychological Association [3] shows that more than half of the American population report that they recognize when they are feeling stressed; however, less than 1 in 3 report successfully managing their stress levels.

For most people, stress may be perceived as such a minor problem that it does not require any treatment seeking or professional assistance [4]. However, major savings in health care costs can be achieved by reducing stress levels or eliminating some of the subjective health complaints. Although the psychobiological mechanisms remain elusive, stress is a risk factor for a wide range of mental and physical health problems, such as cardiovascular disease [5], diabetes [6], and depression [7]. In fact, of all the health conditions predicted by the World Health Organization as having the greatest disease burden by 2030 [8], many have a common contributing underlying factor of stress as either causing or exacerbating disease. Moreover, as many as 40% to 50% of work-related illnesses are related to stress [9,10]. Consequently, it is important to reduce stress in the general population, but this requires scalable interventions with a potentially high reach.

Systematic reviews demonstrate that face-to-face stress-reduction interventions are effective for reducing stress and various health problems [11-14], but their scalability is limited. On the other hand, eHealth interventions have the scalability potential to reach the general population; however, only a small number of studies have focused on stress reduction. Research has mostly focused on posttraumatic stress [15] or stress as a component in interventions primarily aimed at other health problems, such as diabetes [16] or alcohol use [17].

### Web-Based Stress-Reduction Interventions

Most eHealth interventions for stress in the general population have been evaluated in workplace settings and have shown varying results. The earliest studies have documented intervention effects for anxiety and depression [18], stress responses and job satisfaction [19], and beneficial psychophysiological effects on stress [20]. More recent studies have failed to find any effects on stress [21,22], and a few studies that compared Web-based versus therapist-supported stress management interventions demonstrated only short-term and small effects on stress [23,24].

Studies outside of the workplace setting have shown more unequivocal results. Two studies reported improved outcomes for Web-based family or parental stress interventions [25,26], whereas 2 other studies found reduced health distress among participants with various chronic diseases [27,28]. Another study that recruited participants through the Internet and newspaper articles also observed greater improvements in the treatment group [29].

A few studies have also evaluated the impact of stress management as an add-on component to existing eHealth interventions. These studies, however, showed varying results just like Web-based stress-reduction interventions in workplace settings. Christensen et al [30] did not find any additional contribution of stress reduction for depression, whereas Richards et al [31] found only short-term effects of adding stress management for panic disorder. Prochaska and colleagues [32] demonstrated that the add-on of a tailored component to a brief health risk intervention increased the number of participants that were effectively managing their stress.

### Mechanisms of Change

One reason why findings on Web-based interventions for stress are inconsistent, may be the “black box” phenomenon, or lack of understanding as to how and why some interventions work or do not work. Therefore, it is important to investigate the role of potential mediators and moderators of treatment effects. In this study, the Web-based stress-reduction intervention made use of 2 central intervention components—mindfulness and procrastination—both of which are associated with stress.

Mindfulness involves being in the present moment and accepting thoughts and feelings as they occur in a nonjudgmental way [33]. A meta-analysis has shown that mindfulness can have a broad range of health benefits [34] and that mindfulness-based stress-reduction interventions are generally effective [12]. It appears that mindfulness mediates the effect of interventions on stress [35] and it is associated with higher levels of self-regulation [36] which can facilitate deliberate actions to regulate behavior [37] and lessen avoidant coping [38,39]. The latter is a form of procrastination that is characterized by a voluntary extension of the temporal sequencing between an intended course of action and goal-directed behavior, despite one’s expectation of being worse off than before the delay [40]. Numerous studies have shown the negative effects of procrastination, including its relationship to stress [41]. According to the procrastination-health model, procrastination creates unnecessary stress and delays the onset of health promoting behaviors [42]. It is a strategy that brings immediate, albeit temporary, relief from unpleasant or distressing events [43], but ultimately the event remains unresolved.

Temporal dimensions are clearly important to mindfulness, procrastination, and stress, although few theories explicitly specify changes that occur over time or time as a cause of changes in any of these constructs. For example, the key characteristic of mindfulness is a temporal orientation at the present moment that requires a temporal and attentional shift to a state of awareness. However, the temporal course of changes in mindfulness has not yet been fully explored. Studies on procrastination, on the other hand, have indicated that procrastinators experience less stress early on, but more stress closer to a deadline as compared to nonprocrastinators [44]. More recent studies have investigated temporal changes in procrastination using growth curve approaches, and most suggest that procrastination is characterized by a hyperbolic or quadratic function [45]. It is also reasonable to assume that the development of stress changes over time. Daily hassles or acute experiences of stress (ie, meeting a deadline, car trouble, or negative affect) that typically affect a person within hours on the same day of occurrence, are highly transient and rarely affect a person the next day as major stressful life events [46]. Thus, modeling time as an independent variable is important and allows one to represent change or the dynamic relationships between variables, although it remains elusive as to what kind of developmental trajectories one can expect over the course of time in intervention settings.

### Moderating Effects on Stress

In addition to identifying mechanisms of change over time, one may expect variations in intervention efficacy among participants (eg, not all participants improve). Thus, it is interesting to identify participants who benefit the most or participants for who the intervention shows contraindications. For example, the effects of the Web-based intervention on stress responses and job satisfaction, as mentioned previously, were shown particularly effective among males and younger employees [19]. In terms of stress, demographic differences between participants, such as gender, age, and education, can be expected to have varying intervention effects. The reason is that there are demographic differences in stress and how people manage their stress. In general, women report higher levels of stress compared with men [47]. This can be due to women’s multiple roles [48] or the fact that the roles (eg, caregiving) typically assigned to women are stressful [49]. When it comes to age, people use more problem-focused coping strategies and less avoidance coping strategies as they grow older [50,51]. People also develop the ability to self-regulate emotions when dealing with stress as their age increases [52]. This may explain why researchers have found that adults in their 20s report more perceived stress than those in their 50s [53]. But with education, the picture is less clear. A recent large survey demonstrated that work-related stress is associated with higher education [54], whereas previous studies have shown that lower education is a risk factor for stress [55,56].

### Aims of the Study

This study aimed to test whether treatment was predictive of participants’ initial status and different trajectory changes in stress across time. First, it was hypothesized that participants in the Web-based stress-reduction intervention would exhibit lower stress scores at the end of the treatment compared to the beginning, as measured by log server registrations. Second, it was hypothesized that the intervention would reduce levels of stress as measured by online survey data over a period of 6 months as compared to a control group. The control group was expected to remain at approximately the same stress level throughout the study period. Third, the effect of the intervention was expected to be, at least, partially mediated by mindfulness and procrastination over time. Finally, the effect of the intervention was examined with respect to moderating effects of gender, age, and education on the treatment effect on stress over the study period.

## Methods

### Design

The study was a randomized controlled trial consisting of 2 groups to test the effectiveness of a Web-based stress management intervention. Participants were randomized either to the fully automated Web-based Less Stress (LS) intervention or a waitlist control group to test for the natural course of participants’ levels of stress. No unexpected events occurred after the commencement of the intervention (eg, bug fixes, downtimes, email delivery service failures, content changes). Participants in the control group received the intervention after the final data collection. The trial received its ethical approval by the Norwegian Social Science Data Services (reference number: 26816).

### Participants and Recruitment

The study was a Web-based trial without any face-to-face components as part of the recruitment procedure, intervention, or follow-up. Participants were recruited online through a master’s student’s social network on Facebook. In total, 320 first-degree contacts were invited to participate and forward the invitation to their network (ie, viral recruitment).

Potential participants clicked on a link posted on Facebook and were redirected to an external website containing study information and a consent form. Participants had to confirm that they had read the study information and submit the informed consent before they could proceed to the Web-based baseline questionnaire. Eligible participants were implicitly required to (1) read and understand Norwegian, (2) explicitly state that they were 18 years or older, and (3) fill in their email address.

A total of 326 participants were assessed for eligibility. Sixty-five (19.9%) participants did not provide a (valid) email address, and 2 (0.6%) participants reported being younger than 18 years. These 67 (20.6%) potential participants were excluded before randomization. The final sample size that was randomized consisted of 259 participants.

### Randomization

Every participant had an equal probability of being assigned to either the LS or control group. The allocation ratio was set to 1:1 and a series of zeros and ones were generated for each participant using a random integer generator [57]. Because recruitment was carried out through a private and social online network and participants were potentially identifiable through their email addresses, another research member on the team conducted the randomization procedure. This was done to avoid experimenter biases interfering with the randomization. As an extra precaution, email addresses were concealed during randomization.

### Intervention

**Figure 1:**
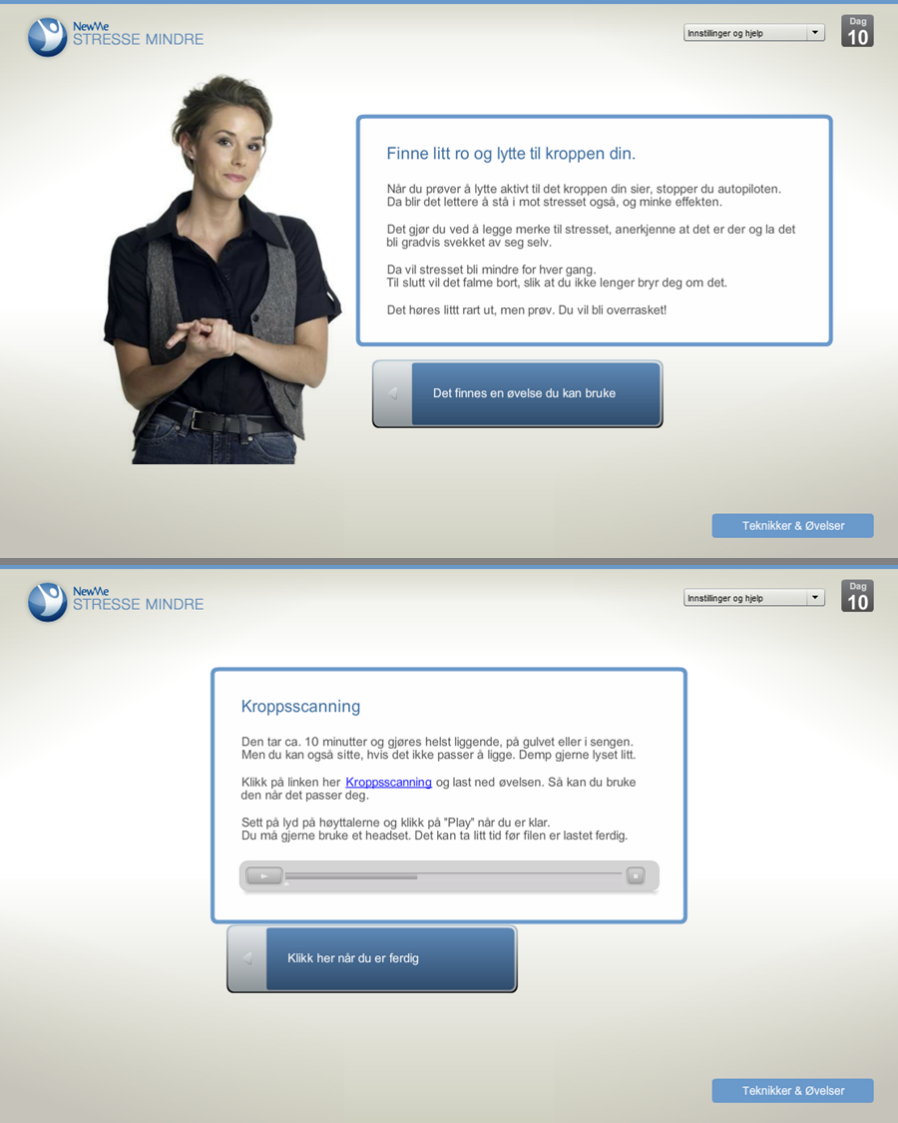
Screenshots from the Less Stress intervention.

The LS intervention is a fully automated and Web-based intervention developed for people who feel stressed or experience a lot of negative emotions (for screenshots, see Figure 1). Its objective is to have users learn about stress, build awareness of sources of stress, and prevent or manage prolonged or high levels of stress. LS uses an eclectic approach and includes evidence-based information and exercises that have been documented to be directly or indirectly effective for stress management, such as mindfulness [12] and metacognitive exercises [58]. The LS intervention consists of 13 sessions over a period of 4 weeks. Every Monday, Wednesday, and Friday, users receive an email with a unique hyperlink. By clicking on the hyperlink, users are directed to a sequence of Web pages that are unique for that particular session. Every session is designed to take approximately 10 minutes to complete. It is a prerequisite that the user completes a session successfully before proceeding to the next session. In this way, the user proceeds through a predetermined therapeutic chronology of sessions with restricted degrees of freedom (ie, tunneled design). For a demonstration, see [59].

Each session contains 2 components. The first component is psychoeducational and addresses some stress-related topics (see Table 1). The second section provides users with techniques, exercises, and homework designed to address the particular topic presented in the psychoeducational section. Psychoeducational information is presented by a young male agent, whereas tasks and exercises are presented by a young female, both accompanied by text designed in such a way “as if they were talking”. The role of the personal computer agent equals that of a domain expert that guides the user. In this way, knowledge and information are represented in a form that is presumably similar to that of a human therapist or expert. Text is presented in short sentences and with a limited amount of text per Web page (approximately 80 words). Techniques and exercises often include audio files (eg, guided instructions for mindfulness exercises) and are often given in the form of home assignments (eg, keep postponing worries to a scheduled time of the day). See Table 1 for a more thorough overview of the contents in LS.

### Data Collection and Measures

Data were collected at baseline (ie, preintervention), and at 1, 2, and 6 months postintervention by means of Web-based surveys. Participants were given 2 weeks to register their responses at each measurement occasion. A reminder email was sent to all nonresponders after 1 week. Log server registrations were also used to collect data on participants in the LS intervention and extracted at the final data collection at 6 months.

Stress was assessed by the stress subscale of the Depression Anxiety and Stress Scale (DASS-S) [60] at every measurement occasion. The DASS-S is a 7-item measure that assesses the severity of the core symptoms of tension (ie, stress) in the past 7 days developed for use with population samples (eg, “I found it difficult to relax”). In the current study, the Cronbach alpha coefficients were .87, .89, .90, and .89 for baseline, 1, 2, and 6 months, respectively. The DASS-S was the primary outcome for the main analyses.

Stress was also assessed in the LS intervention by means of log server registrations as part of the regular intervention. This scale (constructed by the intervention designers by compiling items from several stress measures) consisted of 20 items, such as “I often feel I have too much to do” and “I often set too high personal goals” measured on a 5-point Likert scale (1 = strongly disagree, 5 = strongly agree). The Cronbach alphas were .92 and .93 in sessions 1 and 13, respectively.

The Mindful Attention Awareness Scale (MAAS) [36] assesses the frequency of being aware of what is occurring in the present moment at each repeated measurement. The MAAS is a 15-item scale that was reduced to 10 items for this study. One item was dropped (“...do jobs or tasks automatically, without being aware...”) because it was very similar in Norwegian language to another item that was retained (“...running on automatic, without much awareness...”). Four other items were dropped because the intervention was not developed to tap into these (ie, breaking things, forgetting a person’s name, mindless snacking, and excessive goal focus). The Cronbach alphas in this study were .90, .91, .93, and .92 for each measurement occasion from baseline throughout 6 months, respectively.

Procrastination was measured by the procrastination subscale of the Melbourne Decision Making Questionnaire (MDMQ-P) [61] at each measurement occasion. The MDMQ-P is a 5-item measure of the tendency to avoid decision making (eg, “When I have to make a decision, I wait a long time before starting to think about it”). Its Cronbach alphas were .92, .92, .93, and .94 for baseline, 1, 2, and 6 months, respectively, in this study.

### Statistical Methods

An alpha level of .05 was chosen for all tests and all tests were 2-tailed. To check for baseline differences between groups, *t* tests were used for scales and chi-square (χ^2^) tests for categorical data. All χ^2^ tests that were based on a 2 × 2 contingency table applied the Yates’ continuity correction.

Normality was assessed by means of skewness, kurtosis, and inspection of histograms, with plotted normality curves as visual aids, separately for each treatment group. Skewness was ≤1.43 for the LS group and ≤1.04 for the control group. Kurtosis was ≤4.04 and ≤1.77 for the LS and control group, respectively. This indicates moderate skewness and kurtosis; thus, it was decided not to perform any transformations on data in interest of interpretability.

There were no concerns about violation of homogeneity of variance or variance-covariance matrices with *F*_max_ ratios ≤1.25. Two participants had excessive *z* scores of ±3.29 (*P*<.001, 2-tailed test) on stress at 1 month in the imputed datasets 1 through 5; however, both participants were retained in the dataset as the influence of outliers on mean scores was less than 1.11% after trimming the means by 5%. There were no multivariate outliers as tested by the Mahalanobis distance (*D*) separately for the LS (*D*_6_≤19.83) and control group (*D*_4_≤13.72) with *P*<.001.

There were 113 (43.6%) participants that participated on all measurement occasions; hence, many participants had missing data. Thus, a 2-group multiple imputation (MI) procedure was applied to construct 5 complete datasets for the main analyses (ie, data were imputed separately for the LS and control group) [62]. Auxiliary demographic variables, such as gender, age, education, and intervention adherence, were included in the imputation model to avoid suppressed correlations. Intervention adherence was included in the imputation model for the LS group only. Otherwise, the imputation model was identical for both groups.

Data were longitudinally nested within 2 hierarchical levels, in which time was nested within participants and defined as level 1, whereas participants were defined as level 2. Level 1 variables included the repeated measures of stress, mindfulness, and procrastination, and were measured at baseline and 1, 2, and 6 months. Level 2 variables included demographics and treatment assignment, and were only measured at baseline. All continuous predictors and covariates were centered on the grand mean before modeling.

A series of multilevel models with maximum likelihood estimation were run to analyze the main treatment effect, multiple mediation, and moderation analyses. The overall fit of the models was evaluated by the Akaike information criterion (AIC) and –2 log likelihood (–2LL) on a smaller-is-better basis. Moreover, comparison of nested models was also evaluated formally by a test of differences in –2LL over the difference in degrees of freedom by using an ordinary χ^2^ distribution. A significant difference indicates that the model with the lowest –2LL value fits data better. Analyses were run in SPSS version 20; however, SPSS does not provide pooled model fit indexes in mixed models. Therefore, the median model fit indexes of the 5 imputed datasets are reported. Finally, a pseudo-multivariate coefficient of determination (*R* ^*2*^) was calculated to account for the variation between participants in the final main, mediation, and moderation models. Analyses were also conducted separately with available case analysis. Both procedures produced similar results; thus, only data for the imputed sets are reported.

**Table 1:** Overview of program sessions in the Less Stress intervention.

Session	Description	Psychoeducational content	Techniques and exercises
1	Introduction	Information about program, program structure, and a test of stress levels.	
2	What is stress?	How modern everyday life can affect our stress levels and explanation of stress as a reaction to real or perceived threats to one’s physical and mental well-being.	Mindfulness breathing exercise to help become grounded in the present moment. Homework: Practice mindfulness breathing.
3	What makes you stressed?	Problem-focused vs emotion-focused coping for stress management.	Practical tips on taking care of your economy and worry postponement.
4	Stress and emotions	Debunking the myth of “positive stress.” A bit of stress is not dangerous, but all stress is essentially negative and interlinked with negative emotions.	Mindfulness breathing exercise to help become grounded in the present moment. Homework: practice worry postponement.
5	Physical activity, stress, and mood	Physical activity has an immediate impact on mood and acts as a buffer against stress. Learning detached mindfulness as a way of relating to negative thoughts and feelings.	Engage in physical activity. Practice detached mindfulness by means of the “Clouds in the sky” exercise.
6	Causes of stress	The difference of big stressors (eg, wedding, pregnancy, a new job) and everyday stressors or concerns (eg, noisy neighbors, parking ticket, a dreaded phone call). Identifying everyday stressors and finding ways to deal with these.	Strategies for dealing with everyday stressors: (1) avoidance—avoid situations that cause you stress or negative emotions; (2) change—if avoidance fails, adjust the situation such that the cause of stress dissipates; and (3) reattribute—if changing fails, think of the positives that come out of the stressful situation.
7	Procrastination	Procrastination as a source of stress if delaying all things that are difficult or unpleasant, and how these things can grow into big stressors.	Detached mindfulness: “Tiger task” exercise for relating to difficult or unpleasant situations, thoughts, or feelings.

8	Why do we procrastinate?	Reasons why we procrastinate (1) lack of commitment, (2) lack of motivation if goals are distant, and (3) anxiousness. Identifying personal reasons for procrastination.	Mindfulness: “Gong gong” exercise to help become grounded in the present moment and relaxing by focusing on an external sound.

9	Optimism	Optimism leads to less stress, but too much optimism (ie, being overoptimistic) can cause more stress. Beware of situations that require predicting how much time things take and how much things cost; we almost always underestimate.	Passenger train metaphor - alternative to the “Clouds in the sky” exercise. Negative (and positive) thoughts come and go, just stand by and watch your thoughts pass by.


10	Work-related stress	Explains the “demand, control, and support” model of work-related stress.	Find a balance between demands, control, and support at work. Mindfulness: “Body scan” exercise to increase bodily awareness of how one is doing and become more present in the moment (ie, progressive muscle relaxation).
11	Time management	Everybody has the same amount of time, but some are better at doing 1 thing at a time.	(1) Make 3 lists of activities you have to, ought to, and can do; (2) prioritize and organize each of your lists; and (3) Start with the “have to” list and fill in with items from the “ought to” list. Still time to spare? Fill in with items from the “can do” list without being overoptimistic.
12	Goal management	Reassess your personal goals and get rid of unrealistically high goals (eg, weight loss, career ambitions, personal appearance) that cause a constant feeling of guilt or stress.	Mindfulness: “Body scan” exercise to increase bodily awareness of how one is doing and become more present in the moment (ie, progressive muscle relaxation).
13	Conclusions and summary	Test of your stress level.	

## Results

### Subject Characteristics

**Figure 2:**
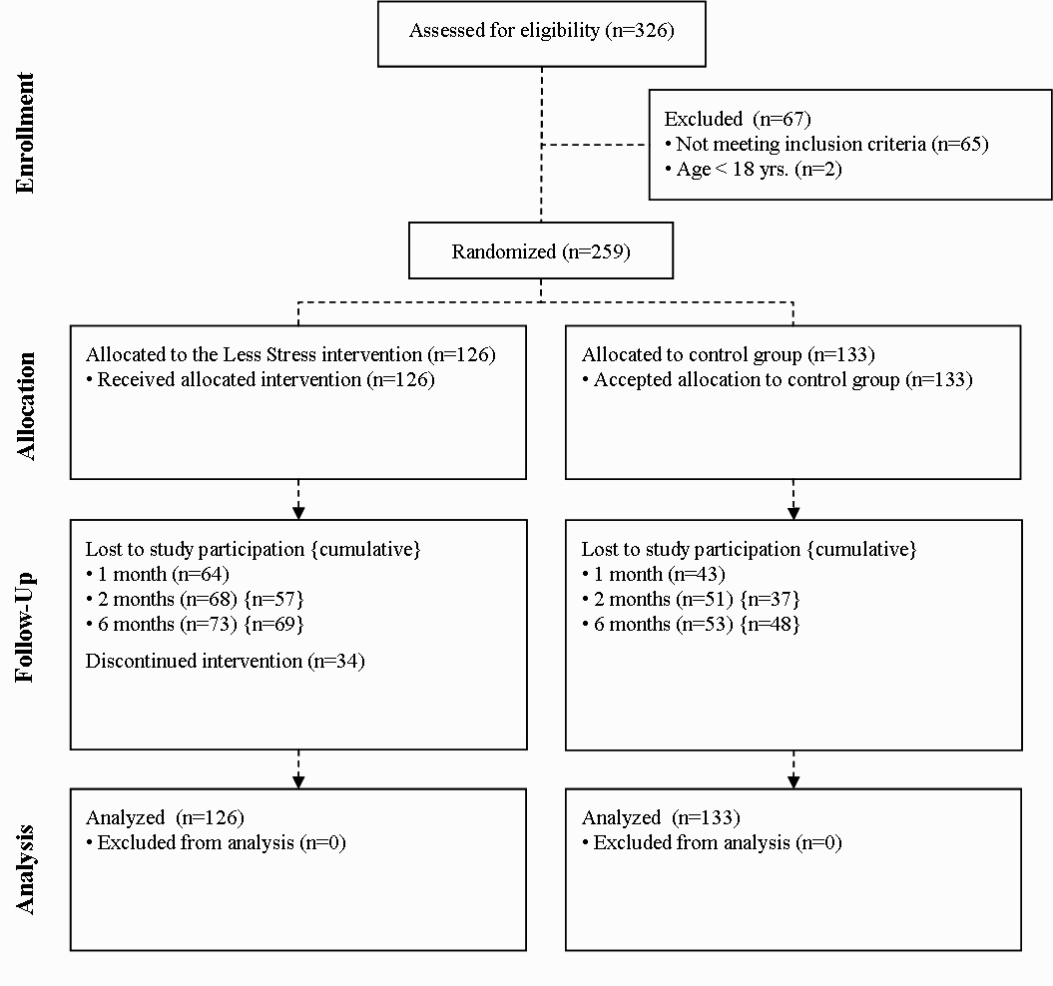
Flowchart of participants.

The flow of participants is depicted in Figure 2. A total of 259 people were eligible for participation and randomized to either the LS intervention or control group. A total of 34 (27.0%) participants in the intervention group discontinued study participation or intervention. Most did not give any reasons for discontinuation, but a few people mentioned reasons such as mail delivery failure (n=2), lack of time (n=2), pregnancy (n=1), too extensive to participate (n=1), and too much to do at work (n=1). Only 3 people provided reasons as to why they discontinued the LS intervention, ie, lack of time (n=2) and too extensive (n=1). Cumulative losses (ie, loss to follow-up on at least 1 previous follow-up) are shown in curly brackets in Figure 2. Note that participants who discontinued the LS intervention were approached for data collection, although 26 (76.5%) of the 34 intervention dropouts were also lost to follow-up.

There were no significant differences between participants in the LS and control group at baseline (Table 2). However, there were more women (76.0% vs 24.0% males, *P*<.01) and participants with ≤1-3 years of college or university education (59.1% vs 40.9% ≥4-5 years of college or university education, *P*<.01) in the total sample. Most were not acquainted with the recruiter (80.3% vs 19.7% acquainted, *P*<.01) indicating that viral recruitment through Facebook was successful in reaching participants beyond the researcher’s first-degree contacts.

**Table 2:** Baseline sample characteristics by group, Less Stress (LS) intervention and control.

Characteristic		LS (n=126)	Control (n=133)
**Gender, n (%)** ^**a**^			
	Men	31 (24.6)	30 (22.6)
	Women	95 (75.4)	98 (73.7)
**Education, n (%)**			
	≤1-3 years in college or university	77 (61.1)	76 (57.1)
	≥4-5 years in college or university	49 (38.9)	57 (42.9)
Age (years), mean (SD)		32.0 (9.6)	33.2 (9.9)
**Acquainted, n (%)** ^**a**^			
	No	97 (78.2)	107 (80.5)
	Yes	27 (21.4)	24 (18.0)
**Psychological variables, mean (SD)** ^**b**^			
	Stress	6.9 (4.7)	7.5 (4.7)
	Mindfulness	34.1 (8.2)	34.1 (9.0)
	Procrastination	18.3 (7.6)	17.1 (8.1)

^a^Numbers and percentages adjusted for missing values.

^b^Higher scores indicate higher levels of stress, mindfulness, and more procrastinating behaviors.

### Attrition and Missing Data

The number of respondents to the follow-up surveys in the total sample were 152 (58.7%), 140 (54.1%), and 133 (51.4%) for 1, 2, and 6 months, respectively. The number of respondents within the LS group was 62 (49.2%), 58 (46.0%), and 53 (42.1%) across the 3 follow-up measurements, and 90 (67.7%), 82 (61.7%), and 80 (60.2%) in the control group. Between-group differences in dropout rates at 1-month (χ^2^_1_=8.4, *P*=.004), 2-month (χ^2^_1_=5.7, *P*=.017), and 6-month (χ^2^_1_=7.8, *P*=.005) follow-up, were significant. Hence, selective attrition is a potential problem regarding the interpretation of levels of stress over the study period. Further, it turned out that the proportion of missing data in the total sample ranged from 0% to 3.5% at baseline and from 42.9% to 52.1% at follow-up (ie, missing data due to item or wave nonresponse). Despite this, Little’s overall test of randomness indicated that the distribution of missing data was not predictable for the LS group (χ^2^_145_=172.2, *P*=.06) and that data for the control group also could be classified as missing completely at random (χ^2^_154_=158.3, *P*=.39).

Due to these potential problems, the effects of selective attrition on means, variances, and relationships among variables used in subsequent longitudinal analyses of treatment effects were assessed, following the procedure described by Goodman and Blum [63]. Because there was little attrition in the study from 1 to 6 months, selective attrition was assessed on the basis of study dropout from baseline to 1 month. The results showed no mean differences between study dropouts and stayers at baseline (-0.59 ≤* t *≤0.63, all *P* values ≥.53). There were also no significant differences in variances using the normal approximation to chi-square [64] in the total sample to those who stayed (-1.91 < *z* <0.36, all *P* values >.06). In other words, selective attrition did not affect the means or variances. However, testing the relationship among variables with multiple regression analyses on stress in the total sample and stayers separately found gender to be a significant predictor of stress at baseline for the total sample (unstandardized beta coefficient [B]=1.40, *P*=.01), but not among stayers (B=0.63, *P*=.44). An independent samples *t* test revealed that males (mean 5.4, SD 3.7) had lower stress scores than females at baseline (mean 7.7, SD 4.8; *t*_130_=–3.96, *P*<.001). Despite that substantial study dropout led to selective attrition, only the relationship between gender and stress was affected. Thus, we can be confident that, other than gender, participant attrition will not affect the results in this study.

### Intervention Use, Acceptance, and Effect

A total of 126 subjects were registered for the LS intervention, of which 92 (73.0%) engaged with LS (ie, initiated use) and 47 (38.5%) completed all 13 sessions. On average, participants completed 6.82 (SD 5.70) sessions and spent 1 hour and 6 minutes (SD 46) on LS. Time spent on LS was below estimated time needed for optimal adherence per session (13 sessions × 10 minutes per session=2 hours and 10 minutes) as tested by a 1-sample *t* test (*t*_91_=–13.27, *P*<.001).

Of the 49 (38.9%) participants that reported data on intervention acceptance at 1 month, LS seemed well received among most. Of these, 41 (83.7%) reported that they believed LS to be “useful to me” whereas 8 (16.3%) participants either disagreed or were indifferent with LS being “useful to me.” Thirty-five (71.4%) participants would recommend LS to others, 10 (20.4%) would neither recommend LS to others or not, whereas 4 (8.2%) users reported that they would not recommend LS to others. Moreover, 46 (89.7%) participants agreed that LS was “easy to use.”

The first hypothesis was that participants in the LS intervention would have lower stress scores at session 13 compared to session 1, as measured by log server registrations. A paired-samples *t* test showed that participants in the LS group had significantly reduced their stress level from session 1 (mean 65.74, SD 13.71) to session 13 (mean 51.91, SD 13.12; *t*_46_=7.54, *P*<.001) as measured by the test of stress levels in the intervention. This equals a large effect size (Cohen’s *d*=1.10).

### Main Effects Analysis

Tables 3 and 4 present the uncentered correlations among study variables from level 1 (ie, repeated measures) and level 2 (ie, participants), respectively. Separate correlation tables for level 1 and level 2 variables were included to avoid aggregation or disaggregation of data. As can be seen in Table 3, all measures of stress, mindfulness, and procrastination correlated significantly at each measurement occasions (all *P* values ≤.002). Stress correlated negatively with mindfulness and positively with procrastination, and mindfulness correlated negatively with procrastination, as expected.

The main hypothesis concerned the comparison of trajectories in stress levels in the LS group and the control group. It was expected that participants in the LS group would reduce levels of stress over a period of 6 months compared to the control group, which would remain at approximately the same stress level throughout. The main effects from the multilevel regression analysis of stress levels are presented in Tables 5 and 6. Model 1 with the repeated measures only indicates that average levels of stress vary significantly across participants and over time. The intraclass correlation coefficient (ICC) was 0.34. This means that 34% of the variation in stress levels is attributable to interindividual differences. In other words, stress varies (naturally) over time for most participants; however, substantial proportions of the variation in stress levels can be attributed to differences between participants over time.

**Table 3:** Correlations among level 1 variables.

Measure	1	2	3	4	5	6	7	8	9	10	11	12
1. Stress baseline	—											
2. Stress 1 month	.32	—										
3. Stress 2 months	.35	.35	—									
4. Stress 6 months	.40	.39	.37	—								
5. Mindfulness baseline	–.59	–.27	–.23	–.26	—							
6. Mindfulness 1 month	–.28	–.53	–.31	–.35	.41	—						
7. Mindfulness 2 months	–.32	–.41	–.52	–.34	.42	.60	—					
8. Mindfulness 6 months	–.27	–.27	–.26	–.43	.35	.48	.53	—				
9. Procrastination baseline	.47	.21	.23	.22	–.56	–.26	–.31	–.32	—			
10. Procrastination 1 month	.26	.35	.20	.25	–.28	–.47	–.36	–.45	.49	—		
11. Procrastination 2 months	.29	.35	.36	.33	–.33	–.44	–.51	–.46	.48	.62	—	
12. Procrastination 6 months	.24	.35	.24	.39	–.25	–.43	–.39	–.53	.40	.57	.61	—

**Table 4:** Correlations among level 2 variables.

Measure	1	2	3	4
1. Treatment^a^	—			
2. Gender^b^	–.01	—		
3. Age	-06^c^	–.04	—	
4. Education^d^	–.04	.11^c^	.07^c^	—

^a^Treatment: 0 = controls, 1 = LS group.

^b^Gender: 0 = male, 1 = female.

^c^ *P*<.05.

^d^Education: 0 = ≤1-3 years of college/university, 1 = ≥4-5 years of college/university.

**Table 5:** Results of functions of time on stress levels over six months.

Model			1		2		3		4	
Treatment effects			Est	SE	Est	SE	Est	SE	Est	SE
**Fixed effects**										
	Intercept		6.31^d^	0.19	6.70^d^	0.22	6.98^d^	0.26	7.20^d^	0.26
	Linear^a^				–0.18^d^	0.05	–0.60^d^	0.22	–2.49^d^	0.66
	Quadratic^b^						0.07^d^	0.03	1.37^d^	0.43
	Cubic^c^								–0.17^d^	0.05
**Random effects**										
	σ^2^ within participants		10.89^d^	0.60	10.75^d^	0.61	10.66^d^	0.61	10.44^d^	0.58
	**σ^2^ between participants**		5.68^d^	0.79						
		Intercept			6.46^d^	1.08	6.92^d^	1.09	7.05^d^	1.08
		Intercept, slope			–0.21	0.16	–0.28	0.15	–0.31^d^	0.14
		Slope			0.00	0.00	0.01	0.00	0.01	0.00
**Model fit indexes**										
	AIC		5708.19		5693.92		5692.41		5686.56	
	–2LL (χ^2^)		5702.19		5681.92		5678.41		5670.56	
	*R* ^*2*^ (level 1)				0.01		0.02		0.04	

^a^Linear: 0 = baseline, 1 = 1 month, 2 = 2 months, 6 = 6 months.

^b^Quadratic: 0 = baseline, 1 = 1 month, 4 = 2 months, 36 = 6 months.

^c^Cubic: 0 = baseline, 1 = 1 month, 8 = 2 months, 216 = 6 months.

^d^ *P*<.05.

A series of multilevel models with a linear, quadratic, and cubic growth parameter were estimated separately to distinguish the natural or normative development of stress over measurement occasions from the treatment effect (ie, models 2-4 in Table 5). The negative estimate of linear growth in model 2 does indicate that, on average, participants experienced reductions in stress levels over time. A test of differences in model fit between model 2 and model 1 yielded a significant result (Δχ^2^_3_=20.3, *P*<.001). A quadratic function of time was added in model 3 to capture any acceleration or deceleration in the rate of change that might occur over the repeated measurements. Results show that participants’ reductions in stress levels tended to accelerate slightly over time. Model 3 showed an improvement in model fit from model 2 at the 10% level (Δχ^2^_1_=3.5, *P*=.06). The variation within groups over time decreased, the AIC value decreased, and the explained variance increased slightly (ie, *R* ^*2*^) for model 3. Thus, it was decided to retain the quadratic function in further analyses. By including a cubic term in model 4, the rate of change in stress levels decelerated again and provided significant improvements in model fit (Δχ^2^_1_=7.85, *P*=.005). Consequently, treatment effects had to be modeled with a linear, quadratic, and cubic growth parameter.

Model 5 with treatment as level 2 predictor shows that, after controlling for the natural development of stress over time, participants in the LS group had significantly lower stress levels as compared to the control group (see Table 6). A formal test of differences comparing models 5 and 4, demonstrated that model 5 fits data better than model 4 (Δχ^2^_1_=9.4, *P*=.002). However, a test of differences in slopes or developmental trajectories between groups was first examined in model 6 that included cross-level interactions between treatment and normative growth over time. The results show that there was a significant interaction effect between treatment and the linear, quadratic, and cubic growth parameters which suggests that, on average, participants experienced reductions in their stress levels, but that participants in the LS group experienced a different developmental trajectory over and above the natural variation in stress over time (model 6 vs model 5: Δχ^2^_3_=8.0, *P*=.046).

**Table 6:** Results of treatment effects on stress levels over six months.

Model			5		6	
Treatment effects			Est	SE	Est	SE
**Fixed effects**						
	Intercept		7.74^e^	0.31	7.40^e^	0.37
	Linear^a^		–2.49^e^	0.66	–0.81	0.85
	Quadratic^b^		1.37^e^	0.43	0.29	0.56
	Cubic^c^		–0.17^e^	0.05	–0.03	0.07
**Predictors**						
	Treatment^d^		–1.10^e^	0.35	–0.40	0.52
	Treatment × linear				–3.45^e^	1.28
	Treatment × quadratic				2.23^e^	0.84
	Treatment × cubic				–0.28^e^	0.11
**Random effects**						
	σ^2^ within participants		10.48^e^	0.59	10.30^e^	0.57
	**σ^2^ between participants**					
		Intercept	6.83^e^	1.07	6.90^e^	1.06
		Intercept, slope	–0.32^e^	0.15	–0.34	0.14
		Slope	0.02	0.00	0.01	0.00
**Model fit indexes**						
	AIC		5679.12		5677.14	
	–2LL (χ^2^)		5661.12		5653.14	
	*R* ^*2*^ (level 1)		0.04		0.05	

^a^Linear: 0 = baseline, 1 = 1 month, 2 = 2 months, 6 = 6 months.

^b^Quadratic: 0 = baseline, 1 = 1 month, 4 = 2 months, 36 = 6 months.

^c^Cubic: 0 = baseline, 1 = 1 month, 8 = 2 months, 216 = 6 months.

^d^Treatment: 0 = control group, 1 = LS group.

^e^ *P*<.05.

**Figure 3:**
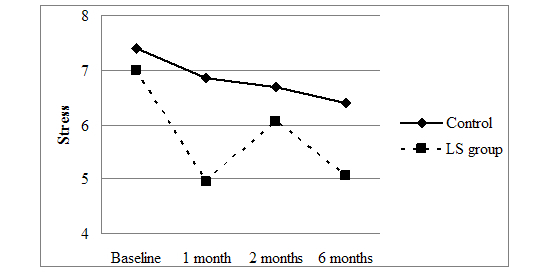
Fitted developmental trajectories for the control and LS group, respectively.

To demonstrate the effect of treatment on initial status and the rate of change over time, Singer and Willett [65] proposed creating prototypical plots. Prototypical plots can be obtained by substituting the values of the treatment variable and time (ie, linear, quadratic, and cubic) variables in the final estimated model (ie, model 6): Y_it_=7.40 + (–0.81)(Time) + (0.29)(Time^2^) + (–0.03)(Time^3^) + (–0.40)(Treatment) + (–3.45)(Time × Treatment) + (2.23)(Time^2^ × Treatment) + (–0.28)(Time^3^ × Treatment), where *Y*_*it*_ is the repeated measure of stress for person *i* at time *t*. The trajectories in Figure 3 demonstrate that the LS group experienced a more immediate and rapid reduction in stress levels as compared to the control group. Although stress levels increased from 1 to 2 months, stress levels returned to the 1-month level by 6 months. In comparison, the control group followed a more modest, steady, and nonsignificant linear decline in stress levels (ie, normative development).

Finally, 3 types of covariance structures relevant and common in studies with repeated measurements were tested. The purpose was to examine how errors were distributed and whether the properties imposed on the covariance structure fit well to the data. The unstructured covariance structure fit the data best as determined by having the lowest information criterion (AIC = 5663.27; compound symmetry AIC = 5678.61 and first-order autoregressive AIC = 5735.48). The assumption of the unstructured error covariance is that all parameters are estimated and allowed to vary freely. In other words, it is the least restrictive covariance structure. Estimates remained largely unchanged with an unstructured covariance structure and are thus not reported.

### Multiple Mediation Analysis

The third hypothesis was that changes in stress levels over time attributed to the LS intervention could be accounted for by changes in mindfulness and procrastination over time. The model-building approach suggested by Bliese and Ployhart [66] was used and a product-of-coefficients strategy was employed to test for multiple mediation effects of treatment on stress via mindfulness and procrastination [67-69]. In other words, the unstandardized beta coefficients for each mediator were multiplied to represent the different indirect effects. Furthermore, the Monte Carlo Method for Assessing Mediation (MCMAM), as described by MacKinnon et al [70], was applied to generate 95% confidence intervals for indirect effects with 20,000 bootstraps [71]. The MCMAM performs reasonably well and can be implemented on the pooled estimates of multiply imputed datasets.

The total effect of treatment on stress was demonstrated previously in model 6 in Table 6 and found to be significant. So, the first step in the mediation analysis was to estimate an unconditional model (ie, model 7a) to test for variation between participants in mindfulness as a mediator over time (see Table 7). The ICC was 0.44, which indicated that average levels of mindfulness vary significantly across participants and over time; hence, a multilevel mediation analysis would be adequate. The next step was to determine the effect of treatment on mindfulness and the fixed functions for time, controlling for procrastination (ie, the second mediator), in a sequence of steps similar to models 2-5 in Tables 5 and 6, although only the final model is reported. Model 7b in Table 7 shows that there was a significant interaction effect between treatment and the linear, quadratic, and cubic growth parameters. This suggests that, on average, participants in the LS group experienced a different developmental trajectory over and above the natural variation in mindfulness over time, after controlling for procrastination.

The effect of treatment on procrastination was tested similarly. The ICC in the unconditional model 8a was 0.51 and suggested that average levels of procrastination vary significantly across participants and over time. Then the effect of treatment on procrastination and the fixed functions for time, controlling for mindfulness was determined. The final model providing the best-fit indexes suggested that treatment had a quadratic effect on procrastination. On average, participants in the LS group did experience a significant decrease in procrastination, although levels of procrastination seemed to increase slightly over time.

**Figure 4:**
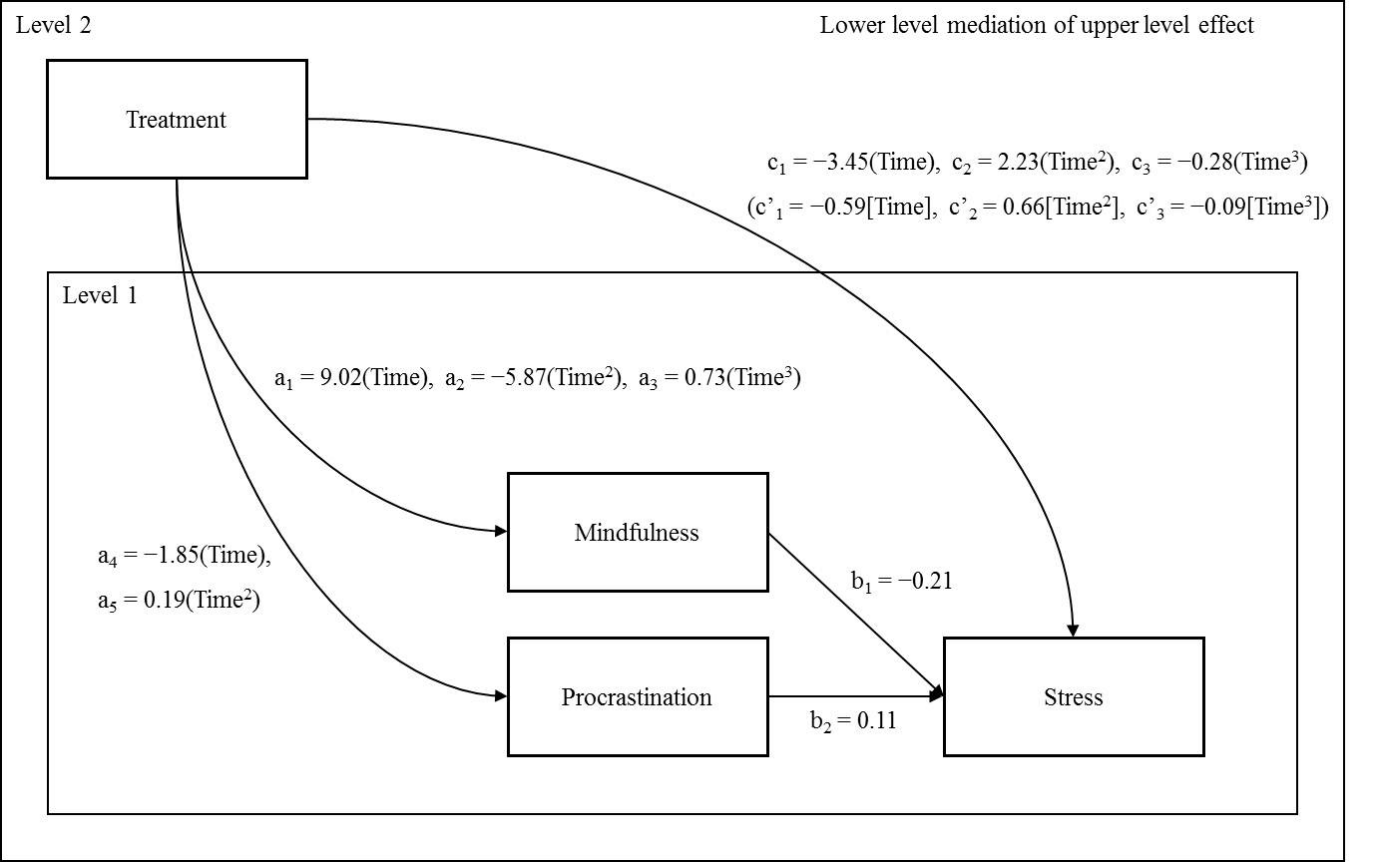
The estimated dynamic multiple mediation model with unstandardized beta coefficients.

Finally, model 9 examined whether the direct effect of treatment on stress was reduced. The results show that mindfulness and procrastination as a set do mediate the effect of treatment on stress levels (see Figure 4). A comparison of the final 2-mediator model (ie, model 9) indicates that this model fit data better than model 6 of the treatment effect (Δχ^2^_2_=279.1, *P*<.001). However, in contrast to model 6 of the treatment effect, an examination of the distribution of errors showed that the best fitting covariance structure was that of compound symmetry (AIC = 5399.26; unstructured AIC = 5403.44 and first-order autoregressive AIC = 5434.60). The assumption of compound symmetry suggests that variances and covariances across the repeated measures were equal.

In simplified terms, the directions of the paths in Table 7 can be interpreted such that the LS intervention leads to greater mindfulness and, in turn, leads to lower stress over time. It also appears that the LS intervention leads to less procrastination, which appears to lead to slightly higher stress over time. An examination of the specific indirect effects in Table 8 indicates that all the specific indirect effects of mindfulness are significant; however, it appears that only the linear specific indirect effect of procrastination is significant. The 95% confidence interval for the quadratic specific indirect effect of procrastination ranged from 0.00 to 0.04 and does not seem to contribute to the indirect effect.

### Multiple Moderation Analysis

The final hypothesis was concerned with examining any moderating effects of gender, age, and education on the treatment effect of the LS intervention on stress scores over time. A reference model of the normative growth and treatment effect was modeled previously (see model 6 in Table 6). Consequently, the first step in the multiple moderation analysis was to model the main effects of gender, age, and education conditional on the normative growth and treatment effects (see model 9 in Table 9). The results show that gender and age had a significant contribution above and beyond normative development and treatment effect on stress. Female participants had higher stress levels than males, whereas stress levels decreased slightly with age. There were, however, no main effects of education. A comparison of model 9 with the addition of the moderators to model 6 of the treatment effects (see Table 6), demonstrated that model 9 performed better (Δχ^2^_3_=17.7, *P*<.001).

The next step was to model the interaction effects between treatment and gender, age, and education, respectively (see model 10 in Table 9). All 3 interaction terms were added simultaneously to adjust for multiple statistical tests and to estimate conditional interaction effects. Results show no interaction effects between treatment and the moderators and no overall improvement in model fit over model 9 (Δχ^2^_3_=6.4, *P*=.09). Finally, a model with interactions between linear growth and all other variables were included to determine whether development was consistent across levels of the other variables (ie, determine time-specific interaction effects; see model 11). Three-way interactions were also included between linear development and other 2-way interactions to test for parallel slopes. Again, there was a significant effect of gender and age on stress over and above the treatment effect, and education also had a significant main effect on stress this time (ie, higher education was related to lower stress). However, although model 11 had improved model fit indexes compared to model 10 (Δχ^2^_6_=24.7, *P*<.001), none of the interactions contributed substantially to the model.

In conclusion, model 9 appears to be the most parsimonious model in which the unstructured covariance structure fit the data best (AIC = 5609.71; compound symmetry AIC = 5678.61 and first-order autoregressive AIC = 5735.48). Estimates remained largely unchanged with an unstructured covariance structure and are not reported.

**Table 7:** The effects of treatment and mindfulness and procrastination on stress levels over time.

Model (dependent variable)			7a (mindfulness)		7b (mindfulness)		8a (procrastination)		8b (procrastination)		Model 9 (stress)	
Treatment effects			Est	SE	Est	SE	Est	SE	Est	SE	Est	SE
**Fixed effects**												
	Intercept		36.04^e^	0.37	34.90^e^	0.60	16.81^e^	0.34	16.15^e^	0.53	7.20^e^	0.30
	Linear^a^				–2.57	1.41			0.42	1.01	–1.51	0.78
	Quadratic^b^				1.97^e^	0.93			0.16	0.63	0.70	0.52
	Cubic^c^				–0.24^e^	0.12			–0.03	0.08	–0.08	0.07
	Treatment^d^				–0.10	0.85			1.48^e^	0.74	–0.79	0.43
	Treatment × linear				9.02^e^	1.95			–1.85^e^	0.54	–0.59	1.25
	Treatment × quadratic				–5.87^e^	1.28			0.19^e^	0.08	0.66	0.84
	Treatment × cubic				0.73^e^	0.16					–0.09	0.11
	Mindfulness								–0.35^e^	0.02	–0.21^e^	0.02
	Procrastination				–0.53^e^	0.04					0.11^e^	0.02
**Random effects**												
	σ^2^ within participants		33.77^e^	1.81	25.39^e^	1.74	21.09^e^	1.10	16.66^e^	1.22	8.57^e^	0.47
	**σ^2^ between participants**		26.44^e^	3.18			21.79^e^	2.46				
		Intercept			20.71^e^	3.26			18.03^e^	2.54	3.00^f^	0.67
		Intercept, slope			–1.54^e^	0.57			–1.20^e^	0.41	–0.08	0.10
		Slope			0.30	0.17			0.16	0.12	0.00	0.00
**Model fit indexes**												
	AIC		6959.34		6687.13		6528.11		6284.20		5402.07	
	–2LL (χ^2^)		6953.34		6661.13		6522.11		6260.20		5374.07	
	*R* ^*2*^ (level 1)				0.25				0.21		0.21	

^a^Linear: 0 = baseline, 1 = 1 month, 2 = 2 months, 6 = 6 months.

^b^Quadratic: 0 = baseline, 1 = 1 month, 4 = 2 months, 36 = 6 months.

^c^Cubic: 0 = baseline, 1 = 1 month, 8 = 2 months, 216 = 6 months.

^d^Treatment: 0 = control group, 1 = LS group.

^e^ *P*<.05.

**Table 8:** Specific indirect effects of mindfulness and procrastination on the effect of treatment on stress over time.

Specific indirect effects		Est	95% CI	
			Low	High
**Mindfulness**				
	Linear	–1.89	–2.83	–1.06
	Quadratic	1.23	0.68	1.84
	Cubic	–0.15	–0.23	–0.08
**Procrastination**				
	Linear	–0.20	–0.35	–0.08
	Quadratic	0.02	0.00	0.04

**Table 9:** Results of multilevel analysis: multiple moderation.

Model			9		10		11	
Treatment effects			Est	SE	Est	SE	Est	SE
**Fixed effects**								
	Intercept		6.80^g^	0.49	6.59^g^	0.58	6.65^g^	0.70
	Linear^a^		–0.81	0.85	–0.81	0.85	–0.83	0.85
	Quadratic^b^		0.29	0.55	0.29	0.55	0.29	0.55
	Cubic^c^		–0.03	0.07	–0.03	0.07	–0.03	0.07
**Predictors**								
	Treatment^d^		–0.45	0.51	–0.09	0.83	–0.58	0.95
	Treatment×linear		–3.45^g^	1.28	–3.45^g^	1.28	–3.27^g^	1.28
	Treatment×quadratic		2.23^g^	0.84	2.23^g^	0.84	2.23^g^	0.83
	Treatment×cubic		–0.28^g^	0.11	–0.28^g^	0.11	–0.28^g^	0.11
	Gender^e^		1.07^g^	0.42	1.37^g^	0.57	1.81^g^	0.71
	Age		–0.04^g^	0.02	–0.08^g^	0.03	–0.08^g^	0.03
	Education^f^		–0.47	0.35	–0.47	0.49	–1.39^g^	0.60
	Treatment×gender				–0.48	0.81	–0.23	1.01
	Treatment×age				0.07	0.04	0.05	0.04
	Treatment×education				0.02	0.72	0.68	0.87
	Linear×gender						–0.16	0.15
	Linear×age						0.00	0.01
	Linear×education						0.34	0.14
	Linear×treatment×gender						–0.09	0.22
	Linear×treatment×age						0.01	0.01
	Linear×treatment×education						–0.24	0.21
**Random effects**								
	σ^2^ within participants		10.29^g^	0.57	10.28^g^	0.57	10.10^g^	0.59
	**σ^2^ between participants**							
		Intercept	6.02^g^	1.00	5.93^g^	0.98	5.81^g^	1.00
		Intercept, slope	–0.25	0.14	–0.27	0.14	–0.19	0.16
		Slope	0.01	0.00	0.01	0.00	0.01	0.01
**Model fit indexes**								
	AIC		5665.48		5665.09		5676.43	
	–2LL (χ^2^)		5635.48		5629.09		5604.43	
	*R* ^*2*^ (level 1)		0.06		0.06		0.07	

^a^Linear: 0 = baseline, 1 = 1 month, 2 = 2 months, 6 = 6 months.

^b^Quadratic: 0 = baseline, 1 = 1 month, 4 = 2 months, 36 = 6 months.

^c^Cubic: 0 = baseline, 1 = 1 month, 8 = 2 months, 216 = 6 months.

^d^Treatment: 0 = control group, 1 = LS group.

^e^Gender: 0 = male, 1 = female.

^f^Education: 0 = ≤1-3 years of college/university, 1 = ≥4-5 years of college/university.

^g^ *P*<.05.

## Discussion

### Principal Findings

Despite the fact that stress is experienced by many people and that stress is a contributing factor to many mental and physical health conditions, few efforts are made to develop and test the effects of interventions for stress. Findings from this study suggest that Web-based interventions can potentially reduce levels of stress. First of all, analysis of log server registrations found large reductions of the LS intervention on levels of stress among intervention completers. Second, treatment was a significant predictor of linear, quadratic, and cubic changes in stress, but not associated with initial status (see model 6 in Table 6). For the linear slope of stress, participants in the LS group showed a faster recovery from stress, although they also had a faster rate of change in stress (ie, increase, quadratic growth) when compared to the control group. Lastly, the LS group had a slower rate of cubic change in stress levels (ie, decrease) than the control group. In other words, despite variations in stress levels, long-term (ie, 6 months) stress levels returned to the level of the immediate short-term effect at 1 month in the LS group. This implies that participants learned ways of managing their stress levels during the course of the intervention that they carried on with them and used to lower their stress levels over time.

There are a limited number of Web-based stress interventions although there is great variability in terms of intervention content and the methods used to evaluate these [18-32]. However, to the extent that there are similarities between some of the studies, it seems that interventions outside of workplace settings (eg, general or family setting) have shown more unequivocally positive findings. It also appears that the single-target interventions are more likely to have an effect on stress [29] than multitarget interventions (eg, dietary behaviors and stress) [23,24]. In some studies, it is not unreasonable to assume that, for example, a small sample may have affected the results [22] or that a high attrition rate was not sufficiently addressed [23]. In contrast to studies which have shown no or only short-term effects, this study has a reasonably high number of participants and data points, addressed attrition and missing data, and examined a single-target intervention in a general setting whose only aim is to reduce levels of stress.

Treatment accounted for approximately 5% of the variation in stress levels across time within participants (ie, 5% of the overall variability in stress is explained by the LS intervention). However, relatively modest treatment effects need not be a problem for eHealth interventions. The distribution of many psychological treatments is concentrated on a large effect for relatively few patients. However, eHealth technologies have the potential to shift this balance. Online consumer behavior suggests that by creating a longer tail in the distribution of eHealth interventions (ie, reaching more users), the market has the potential to substantially increase the collective effect of eHealth technologies [72]. As such, even small and modest changes can be meaningful at the population level. It should, however, be noted that eHealth technologies have yet to reach a large number of users, in particular, the computer illiterate, those with lower incomes, and those without access to the Internet. Even in Norway where the access and use of the Internet is very high in the population, there are digital divides [73]. For example, almost everyone with incomes above NOK 600,000 have Internet access at home, whereas 18% of those with incomes below NOK 200,000 are without Internet access.

This study has not only documented the effect of a Web-based intervention for stress reduction, but also identified its mechanisms of change. As expected and in-line with previous research [35], it turned out that mindfulness mediated the effect of the LS intervention. This is the first study that has examined the relationship between temporal changes in mindfulness and outcomes in an intervention setting. Overall, the results show that mindfulness can be successfully enhanced in Web-based interventions, but that momentary variations in mindfulness can be expected. The LS intervention also led to less procrastination that, in turn, reduced levels of stress as expected based on previous research [41]. More specifically, the results indicate that the LS intervention successfully managed to interrupt the U-shaped (ie, quadratic) pattern of procrastination that can be expected to occur naturally over time [45]. This means the LS intervention led to reduced procrastination that was maintained over time and participants did not, on average, experience the expected increases in procrastination.

Since there often are differences in stress by gender, age, and education, an important finding in this study is that the LS intervention seems to work equally well regardless of these demographic characteristics. In general, female participants reported higher levels of stress and participants that were older reported somewhat lower stress levels, but no demographic characteristics moderated the effect of the LS intervention. This does not mean that there are no psychological moderators of the effects of the LS intervention, for instance, but it may be that the LS intervention can provide a cost-efficient one-size-fits-all approach in terms of demographic characteristics. However, these findings (or lack thereof) should be interpreted with some caution, at least in regards to the result of the analyses of gender.

### Limitations

This study has several limitations. First, the sample in this study was based on viral recruitment on a social networking site (ie, Facebook). Earlier reviews have shown that Internet-based recruitment procedures have faced challenges in recruiting diverse samples [74]. This may be a part of the reason 3 out of 4 participants in this study were women, albeit 80% of those recruited were not acquainted with the female recruiter. This may indicate other explanations of why more women were recruited, such as that more women generally participate in research or that more women are attracted to Web-based self-help interventions [75]. In fact, a recent study did show success in recruiting a diverse sample using Facebook for a randomized controlled trial [76], which further supports the argument for alternative explanations for the gender bias in the recruitment procedure rather than viral online recruitment per se.

There were no reports of negative side effects of using Facebook for participant recruitment in this study; however, the use of social networking sites is an area in need of research and guidelines. Although most were not acquainted with the recruiter, they were acquainted with the person who told them about the study. Thus, a recommendation or study invitation from a friend would have more impact than from a researcher. This also raises ethical issues concerning confidentiality and security in research with peer-to-peer recruitment, but also because websites, such as Facebook, frequently change or update their privacy policies, many of which have been highly controversial. Therefore, it is of utmost importance to carefully consider the recruitment and communication strategies employed via social media, especially for sensitive topics (eg, sexually transmitted diseases), and ensure that participants are redirected to an external website so that the amount of information exchanged on Facebook or similar sites is minimized as in this study or the study by Fenner and colleagues [77] by using advertisements.

The second limitation has to do with selective attrition and missing data. In the LS group, more participants dropped out during follow-up than in the control group. However, the only substantial explanation for study attrition was that more males dropped out most likely because they, in general, had lower stress scores than females. The moderation analyses further confirmed this assumption that inadvertently may have had implications for the power to detect potential interaction effects which is considerably reduced with categorical variables whose categories differ in sample size [78]. However, other than gender, there were no indications that selective attrition or missing data affected the means, variances, or the relationships among variables between those who remained in the study and those who dropped out. Hence, we can be confident about the validity of the results in this study.

The third limitation of this study concerns the mediation analysis. It is becoming more common to investigate complex models in intervention research by using multilevel mediation models, testing for multiple mediators or testing for nonlinear mediation effects [79,80]. In many cases, researchers will assume that there is more than 1 mediator that can potentially affect the outcome of an intervention. Most often, researchers examine mediation with only 1 mediator at a time. Consequently, the effects of multiple mediators cannot be simply examined or compared against each other if researchers examine mediators singly. However, several complications arise when testing for multiple mediators in multilevel models and, unfortunately, there is currently a lack of established procedures or methods for testing indirect effects in multilevel models with multiple mediators where the constituent paths are nonlinear. So, although we may have used the best available methods to date, such as bootstrapping, it is obvious that there is a need to develop a set of recommendations or procedures in this area.

### Conclusion and Future Research

The results from this randomized controlled trial suggest that a Web-based intervention can reduce levels of stress over time and that both mindfulness and procrastination could be important components for inclusion in future eHealth interventions for stress. Future research should make sure to examine the effects of the LS or similar interventions for stress reduction among more male participants and investigate the role of psychological moderators of treatment effects.

## Multimedia Appendix 1

Less Stress intervention screenshots.

## Multimedia Appendix 2

CONSORT EHEALTH checklist V1.6.2 [81].

## Abbreviations

–2LL: –2 log likelihood
AIC: Akaike information criterion
DASS-S: Depression Anxiety Stress Scale–stress subscale
ICC: intraclass correlation coefficient
LS: Less Stress
MAAS: Mindfulness Attention Awareness Scale
MCMAM: Monte Carlo Method for Assessing Mediation
MDMQ-P: Melbourne Decision Making Questionnaire–procrastination subscale
MI: multiple imputation
