# Supplementary material for: Multilevel Growth Curve Analyses of Treatment Effects of a Web-Based Intervention for Stress Reduction: Randomized Controlled Trial
Source: J Med Internet Res. 2013 Apr 22;15(4):e84. doi: 10.2196/jmir.2570 (PMC3636659; doi:10.2196/jmir.2570)
Supplement: Supplementary file 1 [file jmir_v15i4e84_app1.pdf]

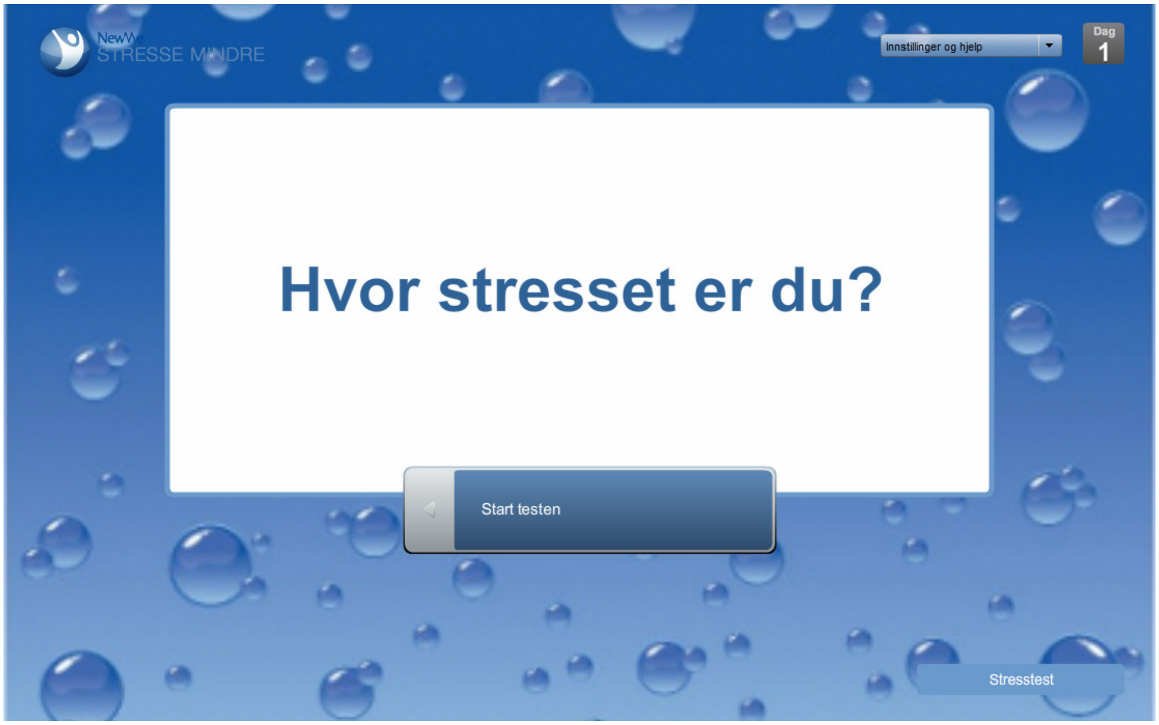
NewMe
STRESSE MINDRE
Innstillinger og hjelp
Dag
1
Hvor stresset er du?
Start testen
Stresstest

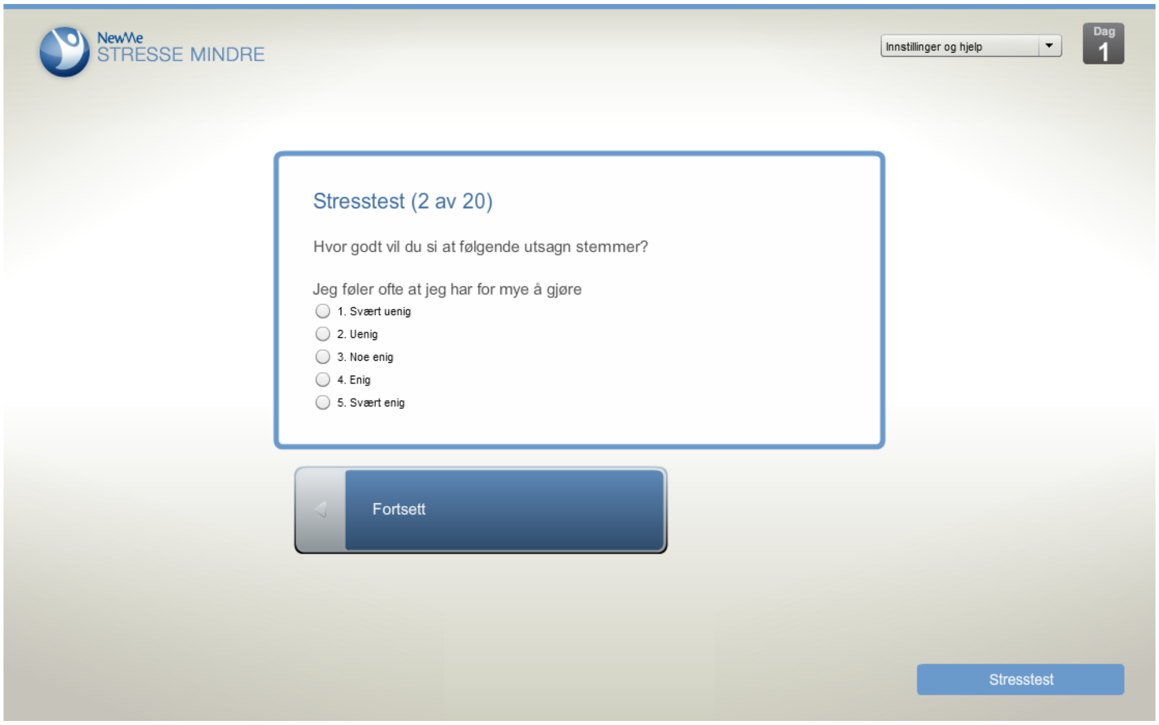
NewMe
STRESSE MINDRE
Innstillinger og hjelp
Dag
1
Stresstest (2 av 20)
Hvor godt vil du si at følgende utsagn stemmer?
Jeg føler ofte at jeg har for mye å gjøre
1. Svært uenig
2. Uenig
3. Noe enig
4. Enig
5. Svært enig
Fortsett
Stresstest

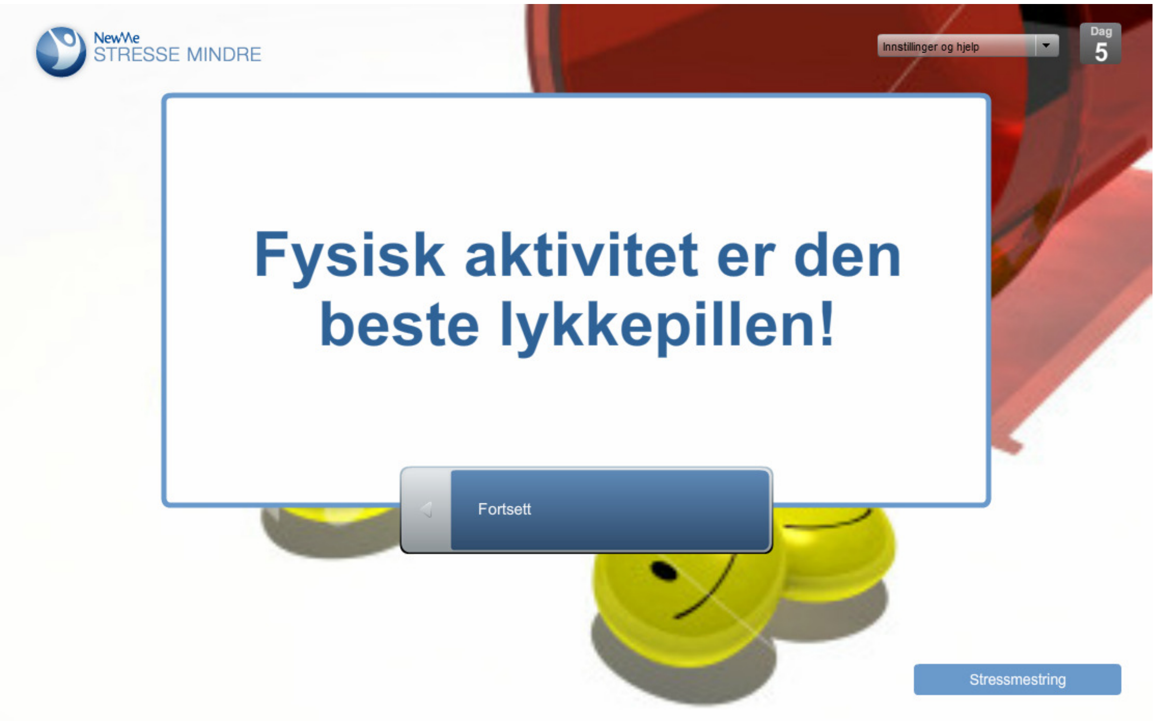
NewMe
STRESSE MINDRE
Innstillinger og hjelp
Dag
5
Fysisk aktivitet er den beste lykkepillen!
Fortsett
Stressmestring

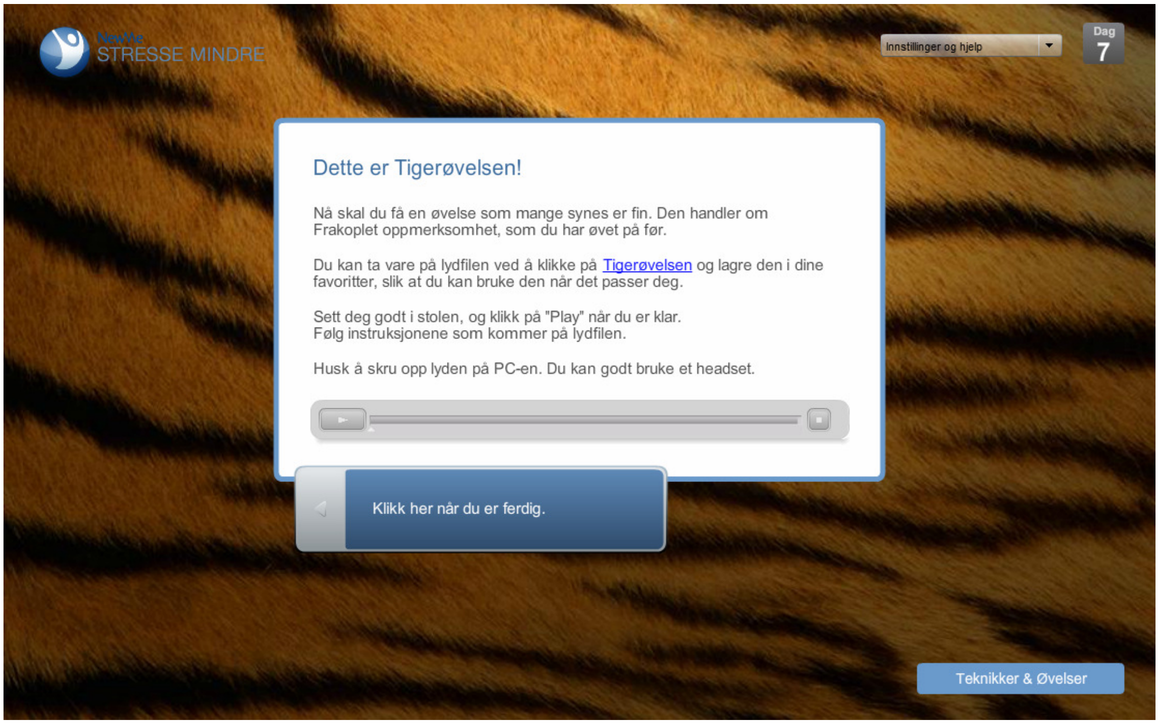
NewMe
STRESSE MINDRE
Innstillinger og hjelp
Dag
7
Dette er Tigerøvelsen!
Nå skal du få en øvelse som mange synes er fin. Den handler om Frakoplet oppmerksomhet, som du har øvet på før.
Du kan ta vare på lydfilen ved å klikke på Tigerøvelsen og lagre den i dine favoritter, slik at du kan bruke den når det passer deg.
Sett deg godt i stolen, og klikk på "Play" når du er klar.
Følg instruksjonene som kommer på lydfilen.
Husk å skru opp lyden på PC-en. Du kan godt bruke et headset.
Klikk her når du er ferdig.
Teknikker & Øvelser

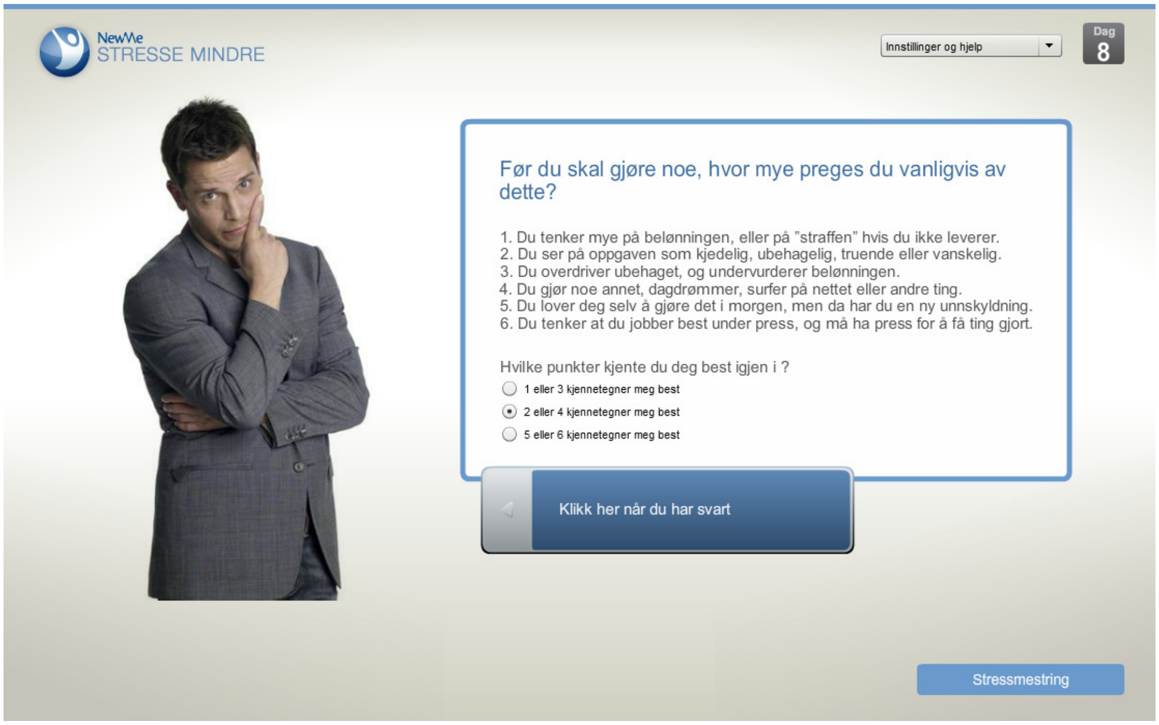
NewMe
STRESSE MINDRE
Innstillinger og hjelp
Dag
8
Før du skal gjøre noe, hvor mye preges du vanligvis av dette?
1. Du tenker mye på belønningen, eller på "straffen" hvis du ikke leverer.
2. Du ser på oppgaven som kjedelig, ubehagelig, truende eller vanskelig.
3. Du overdriver ubehaget, og undervurderer belønningen.
4. Du gjør noe annet, dagdrømmer, surfer på nettet eller andre ting.
5. Du lover deg selv å gjøre det i morgen, men da har du en ny unnskyldning.
6. Du tenker at du jobber best under press, og må ha press for å få ting gjort.
Hvilke punkter kjente du deg best igjen i ?
1 eller 3 kjennetegner meg best
2 eller 4 kjennetegner meg best
5 eller 6 kjennetegner meg best
Klikk her når du har svart
Stressmestring

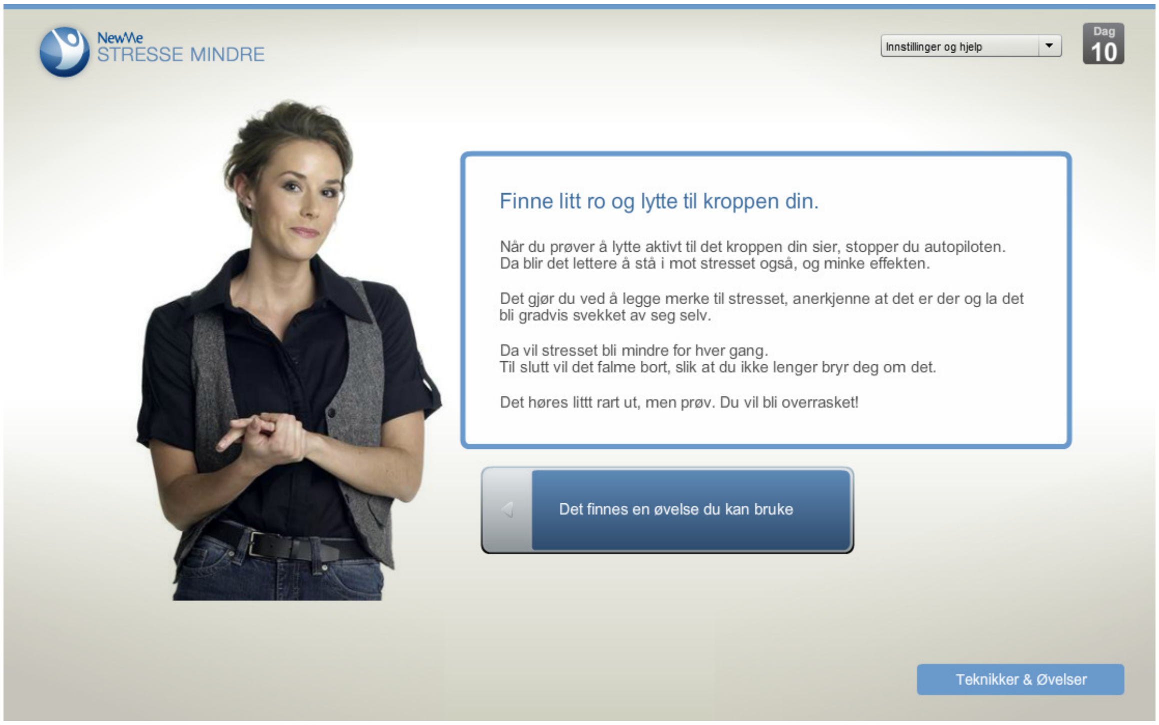
NewMe
STRESSE MINDRE
Innstillinger og hjelp
Dag
10
Finne litt ro og lytte til kroppen din.
Når du prøver å lytte aktivt til det kroppen din sier, stopper du autopiloten.
Da blir det lettere å stå i mot stresset også, og minke effekten.
Det gjør du ved å legge merke til stresset, anerkjenne at det er der og la det bli gradvis svekket av seg selv.
Da vil stresset bli mindre for hver gang.
Til slutt vil det falme bort, slik at du ikke lenger bryr deg om det.
Det høres littt rart ut, men prøv. Du vil bli overrasket!
Det finnes en øvelse du kan bruke
Teknikker & Øvelser

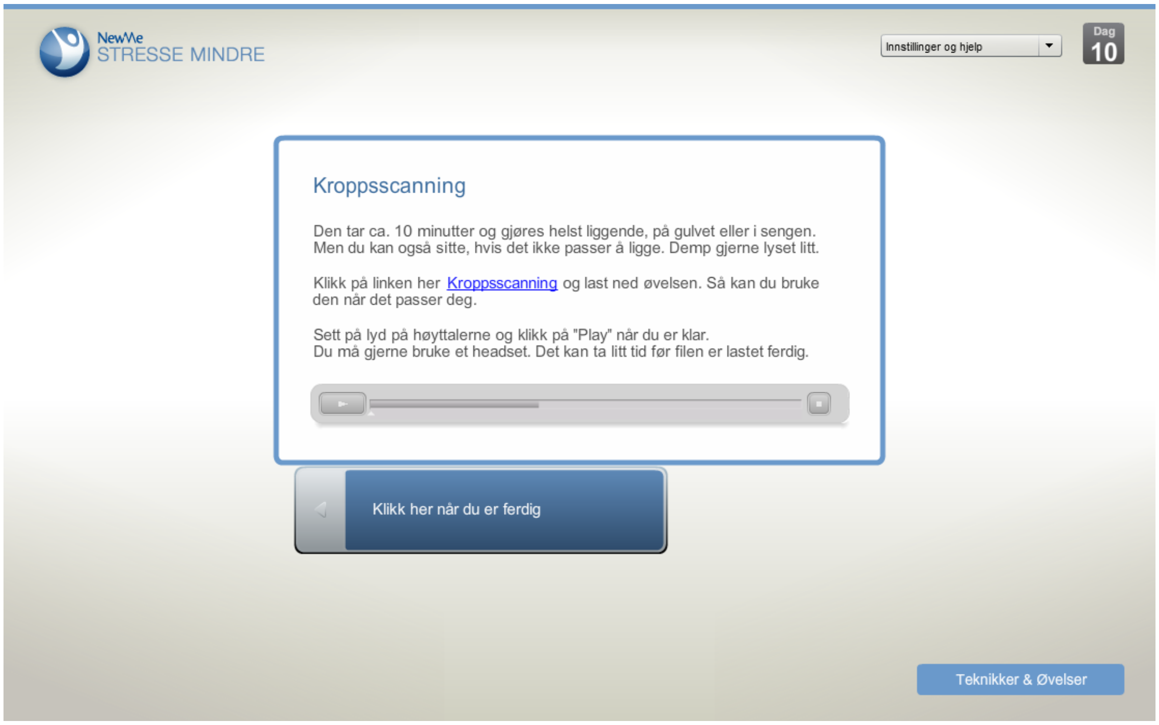
NewMe
STRESSE MINDRE
Innstillinger og hjelp
Dag
10
Kroppsscanning
Den tar ca. 10 minutter og gjøres helst liggende, på gulvet eller i sengen. Men du kan også sitte, hvis det ikke passer å ligge. Demp gjerne lyset litt.
Klikk på linken her Kroppsscanning og last ned øvelsen. Så kan du bruke den når det passer deg.
Sett på lyd på høyttalerne og klikk på "Play" når du er klar.
Du må gjerne bruke et headset. Det kan ta litt tid før filen er lastet ferdig.
Klikk her når du er ferdig
Teknikker & Øvelser
