# Supplementary material for: Multilevel Growth Curve Analyses of Treatment Effects of a Web-Based Intervention for Stress Reduction: Randomized Controlled Trial
Source: J Med Internet Res. 2013 Apr 22;15(4):e84. doi: 10.2196/jmir.2570 (PMC3636659; doi:10.2196/jmir.2570)
Supplement: Supplementary file 2 [file jmir_v15i4e84_app2.pdf]

CONSORT-EHEALTH Checklist V1.6.2 Report

(based on CONSORT-EHEALTH V1.6), available at [http://tinyurl.com/consort-ehealth-v1-6].

2570

Date completed

3/8/2013 8:06:47

by

Filip Drozd

A Randomized Controlled Trial of a Web-Based Intervention for Stress Reduction: Multilevel Growth Curve Analyses of Treatment Effects

TITLE

1a-i) Identify the mode of delivery in the title

Including the term "randomized trial", "randomized controlled trial" or similar is absolutely essential and allows for quick identification such as in the following title: "A Randomized Controlled Trial of a Web-Based Intervention for Stress Reduction: Multilevel Growth Curve Analyses of Treatment Effects".

1a-ii) Non-web-based components or important co-interventions in title

Item 1a-ii was not relevant for the current study as the intervention under investigation was a fully automated web-based intervention. However, all web- and mobile-based interventions should explicitly mention any use of substantial and additional components (e.g. therapist-support or telephone support) such that the results are immediately put into perspective (i.e. the reader is not given any false impressions). For example, there have been reports of studies which do not or just barely mention therapist-support in their manuscript when, in fact, there was extensive contact between therapist and participants.

1a-iii) Primary condition or target group in the title

Item 1a-iii is essential whenever there is a specific health condition or target group under investigation. Otherwise it may not be considered necessary unless there is good reason to emphasize this (e.g. representative population sample).

ABSTRACT

1b-i) Key features/functionalities/components of the intervention and comparator in the METHODS section of the ABSTRACT

Subitem 1b-i is considered such an essential part of randomized trials that key features of interventions and control conditions should be routinely reported such as "The web-based stress intervention was fully automated and consisted of 13 sessions over one month. The controls were informed that they would get access to the intervention after the final data collection."

1b-ii) Level of human involvement in the METHODS section of the ABSTRACT

Item 1b-ii was not relevant as the web-based intervention was fully automated in this study, but the level of human involvement during the intervention should be considered a pre-requisite for abstracts.

1b-iii) Open vs. closed, web-based (self-assessment) vs. face-to-face assessments in the METHODS section of the ABSTRACT

The paper addresses this item in the abstract "Data were collected at baseline, one, two, and six months after intervention onset by means of online questionnaires." However, based on personal experience, the impression is that this is not at all important when reading and evaluating abstracts.

1b-iv) RESULTS section in abstract must contain use data

The results section in the abstract should definitely report the number of participants enrolled or randomized to each group (e.g. "... were allocated to either the stress intervention (n = 126) or the control condition (n = 133)") and mention outcome measures (e.g. "Outcomes were stress, mindfulness, and procrastination..."). It is less important to mention use or uptake of the intervention unless evaluating adherence is the aim of the study. It seems more appropriate to report measures of use in the body text.

1b-v) CONCLUSIONS/DISCUSSION in abstract for negative trials

The current trial showed positive results, however, no changes to primary outcomes or negative trials are also findings(!), and can in many ways be more important than positive findings. Consequently, just as researchers attempt to interpret why findings were positive, they should attempt to examine and interpret why findings were negative or did not show any signs of change (i.e. null-findings).

INTRODUCTION

2a-i) Problem and the type of system/solution

It is important to properly address the problem at hand (e.g. "...according to an international survey, about 75 percent of the general population in developed countries reported feeling stressed on a daily basis ..."), provide a sound theoretical background (e.g. "Temporal dimensions are clearly important to mindfulness, procrastination, and stress, although few theories explicitly specify changes that occur over time..."), and present the state-of-the-art research (e.g. "Studies outside of the workplace setting have shown more unequivocal results. Two studies reported improved outcomes for web-based family or parental stress interventions...") on the type of system or solution under investigation (e.g. web-based interventions). However, intervention details should be provided in greater detail in the methods section.

2a-ii) Scientific background, rationale: What is known about the (type of) system

A state-of-the-art presentation of what is known about the type of system that is the object of study is presented. Context for study or choice of comparator were more appropriately addressed in the methods section.

METHODS

3a) CONSORT: Description of trial design (such as parallel, factorial) including allocation ratio

"This study aimed to test whether treatment was predictive of subjects' initial status and different trajectory changes in stress across time. First, it was hypothesized that subjects in the web-based stress reduction intervention would exhibit lower stress scores the end of the treatment compared to the beginning, as measured by log server registrations. Second, it was hypothesized that the intervention would reduce levels of stress as measured by online survey data over a period of six months, as compared to a control group. The control group was expected to remain at approximately the same stress level throughout the study period. Third, the effect of the intervention was expected to be, at least, partially mediated by mindfulness and procrastination over time. Finally, the effect of the intervention was examined with respect to moderating effects of gender, age, and education on the treatment effect on stress over the study period."

3b) CONSORT: Important changes to methods after trial commencement (such as eligibility criteria), with reasons

No important changes to methods were made during this study.

3b-i) Bug fixes, Downtimes, Content Changes

"No unexpected events occurred after the commencement of the intervention (e.g. bug fixes, downtimes, e-mail delivery service failures, content changes, or other)."

4a) CONSORT: Eligibility criteria for participants

"Eligible participants were implicitly required (a) to read and understand Norwegian, (b) explicitly state that they were 18 years or older, and (c) fill in their e-mail address."

4a-i) Computer / Internet literacy

At this stage in the review process, the current study did not mention computer or internet literacy. Given the fact that the study concerned a web-based intervention, recruitment took place through a social network site, and data were collected online, computer/internet literacy was an implicit eligibility criterion. This is therefore considered more of an ethical concern for discussion rather than a methodological issue.

4a-ii) Open vs. closed, web-based vs. face-to-face assessments:
Item 4a-ii is not subsumed under a separate heading or paragraph in the manuscript, but taken care of and mentioned wherever appropriate under various sections of the paper. It is also explicitly mentioned that "The study was a web-based trial without face-to-face components, neither as part of the recruitment procedure, intervention, or follow-up."
4a-iii) Information giving during recruitment
"Potential subjects clicked on a link posted on Facebook and were re-directed to an external website containing study information and a consent form. Subjects had to confirm that they had read the study information and submit the informed consent, before they could proceed to the web-based baseline questionnaire."
4b) CONSORT: Settings and locations where the data were collected
"Subjects were recruited online through a master student's social network on Facebook."
4b-i) Report if outcomes were (self-)assessed through online questionnaires
"Data were collected at baseline (i.e. pre-intervention), and one, two, and six months post-intervention by means of web-based surveys."
4b-ii) Report how institutional affiliations are displayed
Item 4b-ii not at all important. Describe only if there was reason to suspect that this may have biased the results.
5) CONSORT: Describe the interventions for each group with sufficient details to allow replication, including how and when they were actually administered
5-i) Mention names, credential, affiliations of the developers, sponsors, and owners
"The first author was employed by Changetech AS which developed the Less Stress intervention, at the time of investigation. The third author has a financial interest in the intervention as a shareholder in Changetech AS."
5-ii) Describe the history/development process
Subitem 5-ii is more appropriate for protocols or studies with other aims than that of randomized trials.
5-iii) Revisions and updating
Describe only when relevant.
5-iv) Quality assurance methods
Another item that is more appropriate for protocols or studies with other aims than that of randomized trials. However, providing multimedia content or links to interventions might provide the reader with the opportunity to ensure the accuracy and quality of the information (requires that content is in English though).
5-v) Ensure replicability by publishing the source code, and/or providing screenshots/screen-capture video, and/or providing flowcharts of the algorithms used
Screenshots or links to interventions will be included. However, source code, algorithm flowcharts, etc., are more appropriate for studies documenting the design or development of interventions rather than randomized trials.
5-vi) Digital preservation
The manuscript does conform to this item, see for example:

"American Psychological Association. 2012. Stress in America: Our health at risk. http://www.apa.org/news/press/releases/stress/2011/final-2011.pdf. Archived at: http://www.webcitation.org/6EEhmDYsY."

However, it is believed that it is the nature of the internet that things sometimes perish or cease to exist entirely, even internet archives such as webcitation.org may not stay forever. Consequently, it is more important that the researchers take care of properly documenting and storing their references, links, etc.
5-vii) Access
The paper in its current form does not explicitly address this issue. Subjects were, however, not given any incentives to participate and the context of using the intervention is not possible to verify given that subjects could be using the intervention at home, school, work, or elsewhere.
5-viii) Mode of delivery, features/functionalities/components of the intervention and comparator, and the theoretical framework
An entire section of the manuscript under the methods is dedicated to an description of the intervention.
5-ix) Describe use parameters
"A total of 126 subjects were registered for the LS intervention, of which 92 (73%) engaged with LS (i.e. initiated use), and 47 (38.5%) completed all 13 sessions. On average, subjects completed 6.82 (SD = 5.70) sessions and spent 1 hour and 6 minutes (SD = 46 min) on LS. Time spent on LS was below estimated time needed for optimal adherence per session (13 sessions × 10 minutes per session = 2 hours and 10 minutes) as tested by a one-sample t-test ($t(91) = -13.27$, $P < .01$)."

No specific instructions or recommendations regarding intervention use were given to participants.
5-x) Clarify the level of human involvement
"“Less Stress” (LS) is a fully automated and web-based intervention developed for people who feel stressed or experience a lot of negative emotions."
5-xi) Report any prompts/reminders used
"Every Monday, Wednesday, and Friday, users receive an e-mail with a unique hyperlink."
5-xii) Describe any co-interventions (incl. training/support)
The intervention under study was fully automated. Thus, item 5-xii was not relevant in this case.
6a) CONSORT: Completely defined pre-specified primary and secondary outcome measures, including how and when they were assessed
For example, "Stress was assessed by the Depression Anxiety Stress-Stress sub-scale (DASS-S) [58] at every measurement occasion. The DASS-S is a 7-item measure that assesses the severity of the core symptoms of tension (i.e. stress) in the past 7 days developed for use with population samples (e.g. “I found it difficult to relax”). In the current study, the Cronbach alpha coefficients were .87, .89, .90, and .89 for baseline, one, two, and six months, respectively. The DASS-S was the primary outcome for the main analyses."
6a-i) Online questionnaires: describe if they were validated for online use and apply CHERRIES items to describe how the questionnaires were designed/deployed

6a-ii) Describe whether and how “use” (including intensity of use/dosage) was defined/measured/monitored
See sub-item 5-ix above.
6a-iii) Describe whether, how, and when qualitative feedback from participants was obtained

6b) CONSORT: Any changes to trial outcomes after the trial commenced, with reasons
Not relevant for this study.
7a) CONSORT: How sample size was determined
7a-i) Describe whether and how expected attrition was taken into account when calculating the sample size

The manuscript does not describe sample size or power calculations. There are no existing and agreed-upon ways of calculating sample size for multilevel models, even less so for longitudinal designs with time-level data at level-1. Several softwares exist, but calculate sample sizes very differently. However, sample size was considered adequate given that all data points from the various measurement occasions are used simultaneously in multilevel models. So, with 259 subjects x 4 measurement occasions, there would be more than 1.000 data points per analysis which is far more than is usual with other statistical methods such as t-tests, ANOVAs or multiple regression.

7b) CONSORT: When applicable, explanation of any interim analyses and stopping guidelines

Not relevant for this paper.

8a) CONSORT: Method used to generate the random allocation sequence

"Every subject had an equal probability of being assigned to either the LS or control group. The allocation ratio was set to 1:1 and a series of 0s and 1s were generated for each subject using a random integer generator (www.random.org)."

8b) CONSORT: Type of randomisation; details of any restriction (such as blocking and block size)

"Every subject had an equal probability of being assigned to either the LS or control group." In other words, no restrictions were used during the randomization procedure.

9) CONSORT: Mechanism used to implement the random allocation sequence (such as sequentially numbered containers), describing any steps taken to conceal the sequence until interventions were assigned

The researchers in this study were blinded during the randomization procedure. The randomization was then carried out and participants were assigned to one of the two groups in this study. Only after the randomization were participants informed about group allocation.

10) CONSORT: Who generated the random allocation sequence, who enrolled participants, and who assigned participants to interventions

One team member recruited the participants while another generated the random allocation sequence, assigned the random sequence to participants, and enrolled/registered participants in the Less Stress group for the intervention.

11a) CONSORT: Blinding - If done, who was blinded after assignment to interventions (for example, participants, care providers, those assessing outcomes) and how

11a-i) Specify who was blinded, and who wasn't

It was not possible to blind participants in this study. The national ethical guidelines also requires that participants are informed about possible treatments (e.g. intervention and waitlist conditions) prior to study onset. No blinding of researchers was applied.

11a-ii) Discuss e.g., whether participants knew which intervention was the "intervention of interest" and which one was the "comparator"

Not discussed in the paper. See also sub-item 11a-i above.

11b) CONSORT: If relevant, description of the similarity of interventions

Not relevant.

12a) CONSORT: Statistical methods used to compare groups for primary and secondary outcomes

"A series of multilevel models with maximum likelihood estimation were run to analyze the main treatment effect, multiple mediation and moderation analyses. The overall fit of the models was evaluated by Aikake's Information Criterion (AIC) and -2 log likelihood (-2LL) on a smaller-is-better basis. Moreover, comparison of nested models was also evaluated formally by a test of differences in -2LL over the difference in degrees of freedom using an ordinary $\chi 2$ distribution. A significant difference indicates that the model with the lowest -2LL value fits data better. Analyses were run in SPSS version 20; however, SPSS does not provide pooled degrees of freedom or model fit indices in mixed models. The median degrees of freedom and model fit indices of the five imputed data sets are therefore reported. Finally, a pseudo-R2 was calculated to account for the variation between subjects in the final main, mediation, and moderation models."

12a-i) Imputation techniques to deal with attrition / missing values

"There were 113 (43.6%) subjects that participated on all measurement occasions and, hence, many subjects had missing data. Thus, a two-group multiple imputation (MI) procedure was applied to construct five complete data sets for the main analyses (i.e. data were imputed separately for the LS and control group) [60]. Auxiliary demographic variables such as gender, age, education, and intervention adherence, were included in the imputation model to avoid suppressed correlations. Intervention adherence was included in the imputation model for the LS group only. Otherwise, the imputation model was identical for both groups."

12b) CONSORT: Methods for additional analyses, such as subgroup analyses and adjusted analyses

Not relevant. All planned analyses were carried out and described under the statistical methods section.

RESULTS

13a) CONSORT: For each group, the numbers of participants who were randomly assigned, received intended treatment, and were analysed for the primary outcome

See Figure 1 in the manuscript (i.e. participant flowchart).

13b) CONSORT: For each group, losses and exclusions after randomisation, together with reasons

"Two subjects had excessive Z scores of ± 3.29 ($P < .001$, two-tailed test) on stress at one month in the imputed data sets ($m = 1 – 5$); however, both subjects were retained in the data set as the influence of outliers on mean scores was less than 1.11% after trimming the means with 5%. There were no multivariate outliers as tested by the Mahalanobis distance (D) separately for the LS ($D(6) \leq 19.83$) and control group ($D(4) \leq 13.72$) with $P < .001$". In addition, see the participant flowchart (Figure 1).

13b-i) Attrition diagram

14a) CONSORT: Dates defining the periods of recruitment and follow-up

Start date of recruitment 01/05/2011.
End date of follow-up about 01/12/2011.

14a-i) Indicate if critical "secular events" fell into the study period

Not relevant (i.e. no secular event occurred during the study period. Highly important, however, should only be reported when relevant.

14b) CONSORT: Why the trial ended or was stopped (early)

Not relevant.

15) CONSORT: A table showing baseline demographic and clinical characteristics for each group

See Table 2 in manuscript.

15-i) Report demographics associated with digital divide issues

Demographics such as gender, age, and education were reported. Computer or internet-literacy was not known.

16a) CONSORT: For each group, number of participants (denominator) included in each analysis and whether the analysis was by original assigned groups

16-i) Report multiple "denominators" and provide definitions

Reported whenever possible and relevant.

16-ii) Primary analysis should be intent-to-treat

This paper did not apply the intent-to-treat (ITT) principle. ITT analyses can be quite problematic and provide an easy way out from not having to deal with missing data in more appropriate ways as done in this study. For example, not all participants do not improve as is assumed with ITT analyses. In fact, one of the most common reasons for drop-out is exactly because participants experience improvements. Second, researchers often inadvertently change their research question with ITT analyses without properly acknowledging this. Conventionally, analyses examine the question - If everyone adhered to the regime, would this intervention be effective? - However, ITT analyses examine the issue - Do individuals adhere to this protocol sufficiently to ensure the regime is effective? - that is, ITT analyses confound the question of efficacy and adherence.

17a) CONSORT: For each primary and secondary outcome, results for each group, and the estimated effect size and its precision (such as 95% confidence interval)

R-squares presented in tables 5a - 7 for main analyses.

17a-i) Presentation of process outcomes such as metrics of use and intensity of use

17b) CONSORT: For binary outcomes, presentation of both absolute and relative effect sizes is recommended

Applied where appropriate such as in this example: "The number of respondents within groups was 62 (49.2%), 58 (46.0%), and 53 (42.1%) in the LS group, and 90 (67.7%), 82 (61.7%), and 80 (60.2%) in the waitlist group, across the three follow-up measurements. Between-groups differences in drop-out rates at one ($\chi2$ (1) = 8.35, $P < .01$), two ($\chi2$ (1) = 5.74, $P = .02$), and six ($\chi2$ (1) = 7.76, $P < .01$) months follow-up, were significant."

18) CONSORT: Results of any other analyses performed, including subgroup analyses and adjusted analyses, distinguishing pre-specified from exploratory

No additional or ancillary analyses were conducted in this study.

18-i) Subgroup analysis of comparing only users

19) CONSORT: All important harms or unintended effects in each group

No harms of unintended effects occurred in either group.

19-i) Include privacy breaches, technical problems

Not relevant in this study and should only be described when unintended effects actually occur.

19-ii) Include qualitative feedback from participants or observations from staff/researchers

DISCUSSION

20) CONSORT: Trial limitations, addressing sources of potential bias, imprecision, multiplicity of analyses

20-i) Typical limitations in ehealth trials

The paper addresses three main limitations in the study: (1) recruitment through social networking sites, (2) selective attrition and missing data, and (3) mediation analyses with multilevel models.

21) CONSORT: Generalisability (external validity, applicability) of the trial findings

21-i) Generalizability to other populations

"... we can be confident about the validity and reliability of the results and under the assumption that most real users are likely female, the generalizability of the results are also ensured."

By far, most studies use incidental samples rather than representative samples. This is not necessarily a problem as long as the incidental sample reflects the most likely users of an intervention (i.e. a pragmatic trial). The findings can then be generalized outside of the RCT setting. However, to the extent the study does not reflect its real users outside of the RCT, generalizability is jeopardized.

21-ii) Discuss if there were elements in the RCT that would be different in a routine application setting

22) CONSORT: Interpretation consistent with results, balancing benefits and harms, and considering other relevant evidence

22-i) Restate study questions and summarize the answers suggested by the data, starting with primary outcomes and process outcomes (use)

"Findings from this study suggest that web-based interventions can potentially reduce levels of stress. First of all, analysis of log server registrations found large reductions of the LS intervention on levels of stress among intervention completers. Second, treatment was a significant predictor of linear, quadratic, and cubic changes in stress, but not associated with initial status (see Model 6 in Table 5b)."

22-ii) Highlight unanswered new questions, suggest future research

"Future research should make sure to examine the effects of the LS or similar interventions for stress reduction among more male subjects and investigate the role of psychological moderators of treatment effects."

"...several complications arise when testing for multiple mediators in multilevel models and, unfortunately, there is currently a lack of established procedures or methods for testing indirect effects in multilevel models with multiple mediators where the constituent paths are nonlinear."

Other information

23) CONSORT: Registration number and name of trial registry

"Controlled Trial: International Standard Randomized Controlled Trial Number (ISRCTN): 25619675; http://www.controlled-trials. com/ISRCTN25619675."

24) CONSORT: Where the full trial protocol can be accessed, if available

See item 23 above.

25) CONSORT: Sources of funding and other support (such as supply of drugs), role of funders

Sources of funding was stated in the trial registry and in the acknowledgements in the paper.

X26-i) Comment on ethics committee approval

The manuscript did not include ethical considerations in the methods, but was included after being reviewed.

x26-ii) Outline informed consent procedures

X26-iii) Safety and security procedures

X27-i) State the relation of the study team towards the system being evaluated

"The first author was employed by Changetech AS which developed the Less Stress intervention, at the time of investigation. The third author has a financial interest in the intervention as a shareholder in Changetech AS."
